# Influence of *PTPN22* Allotypes on Innate and Adaptive Immune Function in Health and Disease

**DOI:** 10.3389/fimmu.2021.636618

**Published:** 2021-02-25

**Authors:** Lucas H. Armitage, Mark A. Wallet, Clayton E. Mathews

**Affiliations:** ^1^ Department of Pathology, Immunology, and Laboratory Medicine, University of Florida, Gainesville, FL, United States; ^2^ Immuno-Oncology at Century Therapeutics, LLC, Philadelphia, PA, United States

**Keywords:** PTPN22, PTPN22 620Arg > Trp, type 1 diabetes, cell signaling, rs2476601, autoimmunity, leukocytes

## Abstract

Protein tyrosine phosphatase, non-receptor type 22 (PTPN22) regulates a panoply of leukocyte signaling pathways. A single nucleotide polymorphism (SNP) in *PTPN22*, *rs2476601*, is associated with increased risk of Type 1 Diabetes (T1D) and other autoimmune diseases. Over the past decade PTPN22 has been studied intensely in T cell receptor (TCR) and B cell receptor (BCR) signaling. However, the effect of the minor allele on PTPN22 function in TCR signaling is controversial with some reports concluding it has enhanced function and blunts TCR signaling and others reporting it has reduced function and increases TCR signaling. More recently, the core function of PTPN22 as well as functional derangements imparted by the autoimmunity-associated variant allele of PTPN22 have been examined in monocytes, macrophages, dendritic cells, and neutrophils. In this review we will discuss the known functions of PTPN22 in human cells, and we will elaborate on how autoimmunity-associated variants influence these functions across the panoply of immune cells that express PTPN22. Further, we consider currently unresolved questions that require clarification on the role of PTPN22 in immune cell function.

## Introduction

Almost 1.6 million Americans have Type 1 Diabetes (T1D), an autoimmune disease that results in destruction of the insulin producing β cells in the pancreas and eventually requires exogenous insulin (1). T1D shows familial clustering and concordance rates between monozygotic twins is over 50% indicating that T1D has a strong genetic component (2, 3). It is estimated that up to 88% of the phenotypic variance is due to genetic factors such as predisposing or protective human leukocyte antigen (HLA) haplotypes and SNP-tagged variants (4–6). Of the genetic component of T1D risk, the HLA region, encoding the major histocompatibility complex (MHC) proteins, accounts for approximately 50% of heritable risk (7). The MHC class I (MHC-I) proteins are expressed on all nucleated cells and present antigenic peptides to CD8^+^ T cells while the MHC class II (MHC-II) proteins are primarily expressed on APC subsets and present antigen only to CD4^+^ T cells. The HLA Class II genes, encoding MHC-II, are the major contributing factor of HLA to risk with the DR3 (DRB1*03:01), DR4 (DRB1*04:01/02/04/05/08), DQ8 (DQA1*03:01-DQB1*03:02/04), and DQ2 (DQA1*05:01-DQB1*02:01) haplotypes conferring the greatest risk (7, 8). Indeed, the DR3/4 diplotype confers the greatest risk for T1D development (9, 10). These haplotypes increase risk in a synergistic manner and current research shows they have augmented ability to present T1D autoantigens to T cells, possibly due to alterations in the critical amino acids in the peptide binding pocket involved in which peptides are presented (10–12).

Although the HLA region contributes the bulk of genetic risk for T1D, there have been over 60 non-HLA genetic loci identified that have variants associated with enhanced or reduced risk of T1D (4, 13–22). Of these non-HLA loci, a non-synonymous SNP in *PTPN22* has one of the highest reported odds ratios, ~2, and has been repeatedly confirmed across multiple studies and populations (4, 13, 15, 23–25). Protein tyrosine phosphatase, non-receptor type 22 (PTPN22) is a negative regulator of T cell receptor (TCR) and B cell receptor (BCR) signaling (26, 27). The diabetes-associated SNP in *PTPN22* (*rs2476601*) affects TCR and BCR signaling as well as other adaptive and innate immune cell processes (27–39). The following sections will elaborate the known functions of PTPN22 and its autoimmune-linked/diabetogenic, missense SNP in human cells and how this might contribute to the pathogenesis of T1D. While the primary focus of this review is on human biology, we will emphasize specific areas of murine *Ptpn22* research, where relevant, to highlight key similarities and differences between species.

## Genetic Variation in *PTPN22*

Protein tyrosine phosphatase, non-receptor type 22 (PTPN22) is expressed in leukocytes and is well-known as a negative regulator of TCR and BCR signaling (26, 27). In non-activated T cells PTPN22 directly complexes with C-src tyrosine kinase (Csk) (32, 40, 41). This interaction is enhanced by phosphorylation of PTPN22 on Ser^751^ by PKCa. Further, phosphorylation of this residue increases the half-life of PTPN22 by protecting the enzyme from K48-linked ubiquitination and preventing recruitment of PTPN22 to the plasma membrane (42). During leukocyte activation PTPN22 is recruited to the plasma membrane to limit proximal immune cell receptor signaling. Here PTPN22 interacts with and dephosphorylates Grb2 (43), VCP (44), Vav (32, 44), Zap70 (32, 44), Lck (26, 32, 44), TCRζ (44), CD3ϵ (44), c-CBL (45), EB1 (46), and the p85 subunit of PI3K (47) to downregulate NFAT and reduce IL-2 production and secretion. However, PTPN22 also acts a regulator of other signaling networks (i.e., interferon γ receptor signaling, LFA-1 signaling, and TLR4 signaling) in monocytes, macrophages, dendritic cells, and neutrophils (29, 32, 35). There are multiple non-synonymous SNPs in PTPN22 associated with increased risk or decreased risk of autoimmune diseases (**Table 1**). The minor allele at *rs56048322*, PTPN22^K750N^, influences PTPN22 splicing and appears to cause CD4^+^ T cell hyporesponsiveness that increases risk for T1D (48). The minor allele at *rs33996649*, PTPN22^R263Q^, is a loss-of-function variant with diminished phosphatase capacity that reduces the risk of both SLE (49) and RA (50) (**Table 1**). Here we will examine *rs2476601*. The minor allele has a thymine substituted for a cytosine at nucleotide 1858, *PTPN22^C1858T^*, and encodes a tryptophan instead of an arginine at amino acid 620, PTPN22^R620W^ (**Table 1**). It was first linked to T1D by Bottini et al. in 2004 (51) and the association between *rs2476601* and T1D was quickly replicated (52). This SNP has also been associated with increased risk for multiple autoimmune diseases including rheumatoid arthritis (RA) (28), systemic lupus erythematosus (SLE) (53), Graves’ disease (52, 54), myasthenia gravis (55), primary Sjogren’s syndrome (56), generalized vitiligo (57), Addison’s disease (58), and alopecia areata (59) strongly suggesting PTPN22 regulates immunity.

**Table 1:** Single nucleotide polymorphisms in human *PTPN22*, their analogous mutations in mice, and their disease associations.

SNP	Human (*PTPN22* or PTPN22)		Mouse (*Ptpn22* or PEP)		Effect	Associations
	Major Allotype	Minor Allotype	Major Allotype	Minor Allotype		
*rs2476601*	PTPN22^620R^	PTPN22^620W^	PEP^619R^	PEP^619W^	variable	Increased risk multiple autoimmune diseases
*rs56048322*	PTPN22^750K^	PTPN22^750N^	–	–	alternative splice variant	increased risk T1D (48)
*rs33996649*	PTPN22^263R^	PTPN22^263Q^	PEP^195D:227C^	PEP^195A:227S^	loss-of-function	reduced risk SLE (49) and RA (50)

The SNP, *rs2476601*, lies in the proline-rich c-terminal domain of PTPN22 and interrupts some protein-protein interactions (e.g., interactions with CSK, TRAF3, and PAD4) (30, 35, 51). This is well illustrated in a recent review article (60). To determine the function of the common or major allotype of PTPN22, namely PTPN22^620R^, diverse approaches including knock down or overexpression of *PTPN22* in primary human cells or human cell lines and knock down/out of *Ptpn22*, the mouse orthologue of *PTPN22*, in mice and mouse cell lines, have been used. To study the altered function of the minor allotype of PTPN22, PTPN22^620W^, researchers have again utilized many techniques including comparative studies in primary cells from human PTPN22^620W^ donors vs. PTPN22^620R^ donors, overexpression of PTPN22^620W^ vs. PTPN22^620R^ in primary human cells and human cell lines, transgenic expression of human PTPN22^620W^ vs. PTPN22^620R^ in *Ptpn22* ^−/−^ mice, and introduction of a mutation that is analogous to PTPN22^620W^ in the mouse orthologue, PEP^619W^. Notably, this SNP is also associated with protection from *Mycobacterium tuberculosis*, an infection primarily controlled by T cells and T cell-activated macrophages (61–64). PTPN22 has been described as a negative regulator of multiple stages of danger signal recognition, from the process of T and B cell education, throughout initial detection of microbes, and then T and B cell effector functions. Thus, genetic variation that confers beneficial immunity to a globally-relevant pathogen (*M. tuberculosis*) might lower the threshold for danger signal responses. In murine models of T1D, lack of key macrophage/CD4^+^ T cell effector molecules (e.g., CD154 and CD40) but not all (e.g., IFNγ and IFNγR) prevents autoimmunity in T1D-prone NOD mice (65–67). We propose that the T1D-associated risk allotype of PTPN22 permits excessive innate and adaptive immune signaling in response to aseptic and/or septic stress/danger signals, in turn, driving a type IV delayed hypersensitivity response against pancreatic β cell antigens. The end result is insulin deficient diabetes mellitus. Herein we review the findings that support a pan-leukocyte role for PTPN22 in immune regulation. For the purpose of this review, we will examine the known roles for PTPN22 in innate and adaptive leukocyte signaling pathways and functions in humans as well as supporting data from mouse models. Where data is available we will also discuss how the minor allotype of PTPN22, PTPN22^620W^, influences signaling pathways as well as cellular functions and how these alterations may contribute to the development of T1D.

## PTPN22 Expression

*PTPN22* is expressed in most types of human leukocytes, including CD4^+^ T cells, CD8^+^ T cells, B cells, NK cells, monocytes, macrophages, dendritic cells, and neutrophils. Of these cells, *PTPN22* has the highest expression in activated naïve CD8^+^ and CD4^+^ T cells, followed by NK cells and B cells, with lower levels in monocytes (28, 68). While the non-synonymous SNP at *rs2476601* changes the amino acid sequence, the allelic difference does not modify *PTPN22* expression in most lymphocyte subsets. Peripheral blood mononuclear cells (PBMCs) from PTPN22^620R/W^ donors expressed *PTPN22* mRNA equally from both alleles and this did not vary with gender (69). Upon anti-CD3/anti-CD28 stimulation of PBMCs (simulated activation of the TCR/CD3/CD28 complex), *PTPN22* mRNA expression increased and this rise in expression was equally attributed to both alleles (69). Similarly, *PTPN22* expression levels in PMBC-derived DCs and PBMC are the same in PTPN22^620R/W^ and PTPN22^620R/R^ donors (35).

There are, however, exceptions; PTPN22^620W/W^ donors had 9% lower *PTPN22* expression in naïve CD4^+^ T cells compared to PTPN22^620R/R^ donors but there were no additional differences in *PTPN22* expression in other T cell subsets (47). There is a report showing that PTPN22^620W^ is more susceptible to calpain-1-mediated degradation and that the PTPN22^620W^ protein is less expressed in naïve and memory T cells compared to PTPN22^620R^ (70); yet, this has been disputed by later studies that observed the antibody used to detect PTPN22 had a higher affinity for PTPN22^620R^ versus PTPN22^620W^ (35, 40, 71). *PTPN22* mRNA and protein expression in freshly-differentiated macrophages (so-called M0 or non-polarized macrophages) from PTPN22^620R/W^ and PTPN22^620W/W^ donors was lower than that of PTPN22^620R/R^ donors (38). After M1 polarization of these macrophages (treatment with lipopolysaccharide and IFN-γ to mimic an inflamed septic environment), mRNA and protein expression of *PTPN22* was higher in PTPN22^620R/W^ and PTPN22^620W/W^ donors than PTPN22^620R/R^ donors but there was no difference in M2 polarized macrophages (treatment with IL-4 and IL-13 to generate so-called “alternatively activated macrophages”) (38). For macrophages, these findings are suggestive of a relationship between *PTPN22* allotype and *PTPN22* expression in the context of microbial infections wherein type 1 CD4^+^ T helper response (T_H_1) typified by IFN-γ secretion occur – for example, mycobacterial infections. Overall, allelic differences at *rs2476601* have modest effect on the expression of PTPN22 in human cells that might be associated with observed immune phenomena (e.g., altered susceptibility to mycobacterial infections), but many questions remain unanswered and causality is merely speculative until more complex studies can be completed. While *PTPN22* expression is only modestly influenced by allele, the function of *PTPN22* is measurably altered by *rs2476601*.

## Regulation of T Cell Function by PTPN22 Allotypes

The majority of studies focused on *PTPN22* have investigated how the PTPN22^620^ allotypes influence the composition of the T and B cell compartments and intracellular signaling in T cells and B cells. PTPN22 allotypes have minor effects on T cell composition across immune compartments in humans; there are no differences in total T cells, total CD4^+^ or CD8^+^ T cells, or CD4^+^ or CD8^+^ effector memory T cells when comparing PTPN22^620R/W^ donors to PTPN22^620R/R^ donors (72). Most studies report no differences in most CD4^+^ T cells subsets (i.e., T_H_1, T_H_17, T_H_1T_H_17, T_FH_) (73). However, PTPN22^620W/W^ donors had slightly-increased FOXP3^+^CD4^+^ regulatory T cells(T_regs_) (7.94% vs. 6.76%) compared to donors with the common PTPN22^620R/R^ allotype (74, 75). It has been reported that PTPN22^620R/W^ donors have increased memory CD4^+^ T cells when compared to PTPN22^620R/R^ donors (about 50% vs. 41% respectively) with a concomitant decrease in naïve CD4^+^ T cells (76). EOMES is a T box transcription factor that drives IFNγ secretion by CD4^+^ T cells (73). PTPN22^620W/W^ donors exhibited increased EOMES^+^CD4^+^ T cells compared to PTPN22^620R/R^ donors (~7% vs. ~5%) again with an accompanying decrease in naïve CD4^+^ T cells (73). It is unclear whether *PTPN22* genotype influences naïve CD4^+^ T cell frequency (72, 73, 76). Two studies have reported a trend toward decreased naïve CD4^+^ T cells in PTPN22^620W/W^ donors (73, 76) while a third study reported no difference in naïve CD4^+^ T cells when examining *PTPN22* genotype (72). The study that reported no difference had a low number of subjects (3 in each group) and no subjects that were homozygous for the minor allele (72). The two studies that have reported a difference included more participants [13 per group (73) or ≥22 per group (76)] and included a group homozygous for the minor allele. Differences in study populations may explain the inconsistencies. A study with a larger cohort of all three genotypes (i.e., *PTPN22^1858C/C^*, *PTPN22^1858C/T^*, and *PTPN221858^T/T^*) may be better powered to address whether *PTPN22* genotype influences naïve CD4^+^ T cell frequency.

### Impact of PTPN22 Allotypes on TCR Signaling

**Figure 1:**
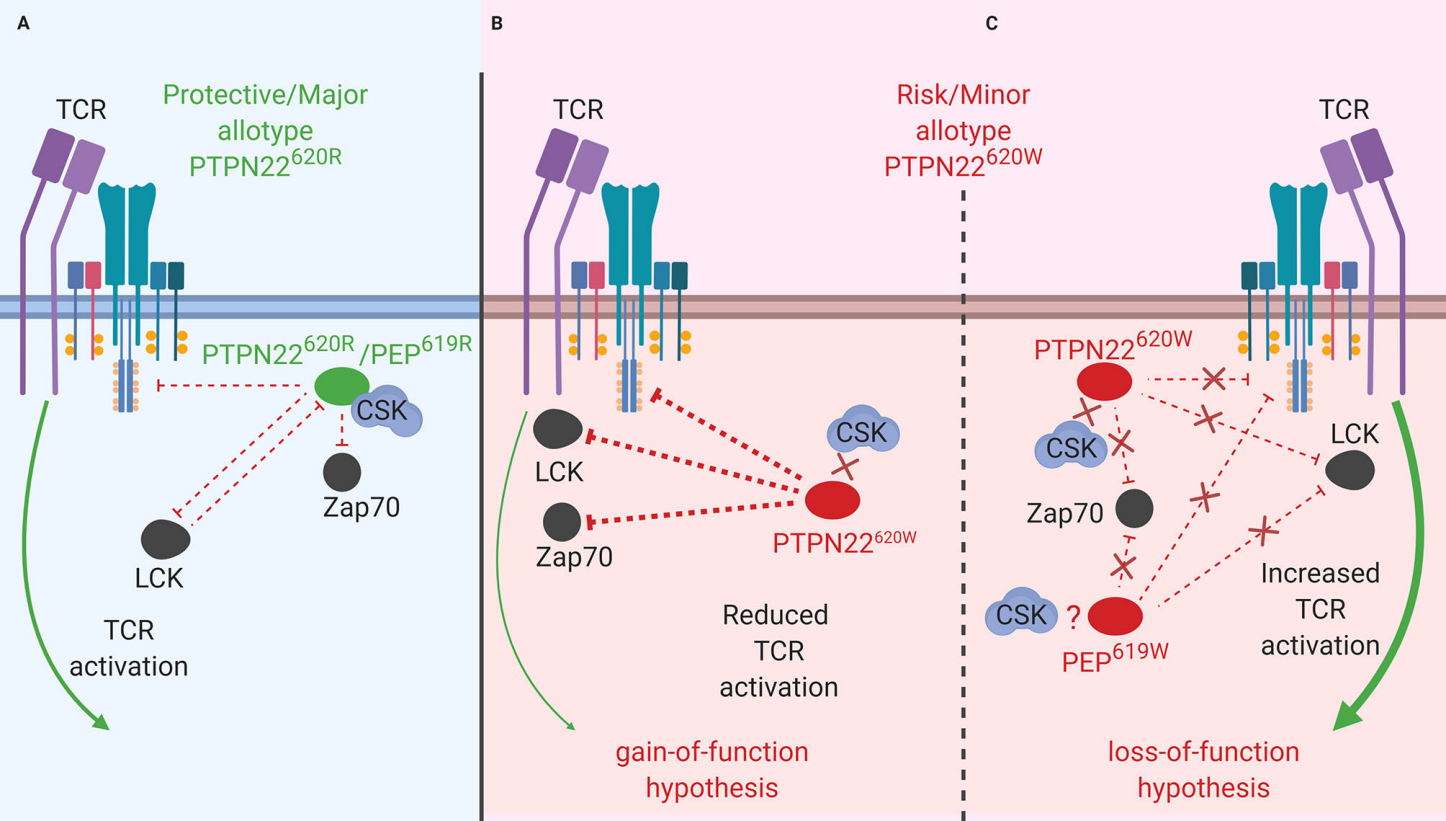
PTPN22 function in T cells. **(A)** PTPN22^620R^ and PEP^619R^ are negative regulators of TCR signaling in T cells where they dephosphorylate/deactivate signaling intermediates and reduce signaling from the TCR to the nucleus. **(B)** The PTPN22^620W^ gain-of-function hypothesis. In this scenario, PTPN22^620W^ is more active and dephosphorylates signaling intermediates at an increased rate compared to PTPN22^620R^. This blunts TCR signaling compared to PTPN22^620R^ and reduces T cell response. **(C)** The PTPN22^620W^ and PEP^619W^ loss-of-function hypothesis. In this scenario, PTPN22^620W^/PEP^619W^ are less efficient at dephosphorylating TCR signaling intermediates compared to PTPN22^620R^/PEP^619R^. This allows more signal from the TCR to reach the nucleus and increases T cell response to TCR stimulation.

**Figure 2:**
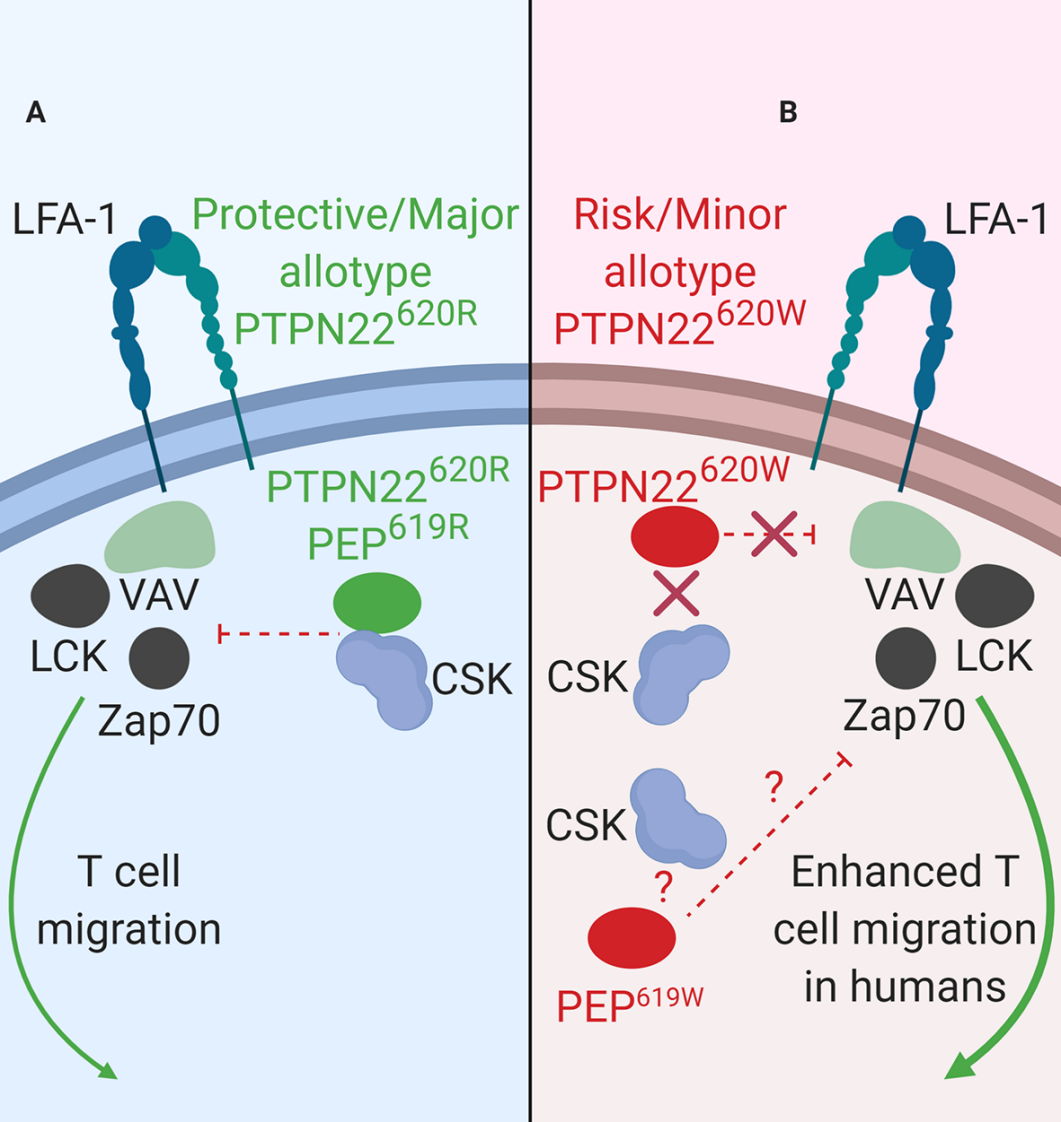
PTPN22 function in LFA-1 signaling. **(A)** PTPN22^620R^ and PEP^619R^ are negative regulators of LFA-1 signaling in T cells. Upon LFA-1 binding of ICAM-1, PTPN22^620R^ and PEP^619R^ associate with CSK, and are recruited to the leading edge in an LCK-dependent manner where they dephosphorylate PTPN22 substrates and inhibit LFA-1 signaling. **(B)** PTPN22^620W^ is a loss-of-function variant in LFA-1 signaling. Upon LFA-1 binding of ICAM-1, PTPN22^620W^ does not associate with CSK and is not recruited to the leading edge. This prevents PTPN22^620W^ from interacting with its substrates and inhibiting LFA-1 signaling. PEP^619W^ has not been studied in this context.

While PTPN22 allotypes have a minor impact on T cell compartment composition, a significant impact on signal transduction in human T cells has been observed. In primary T cells, PTPN22^620R^ is a negative regulator of TCR (26, 28, 43, 77, 78) (**Figure 1A**) and lymphocyte function-associated antigen 1 (LFA-1) (32) signaling (**Figure 2**) while it is a positive regulator of *in vitro* T regulatory cell (T_reg_) induction (33). In T cells, PTPN22^620R^ has been shown to directly interact with Grb2 (43), VCP (44), Vav (32, 44), Zap70 (32, 44), Lck (26, 32, 44), TCRζ (44), CD3ϵ (44), c-CBL (45), CSK (32, 40, 41), EB1 (46), and the p85 subunit of PI3K (47). Studies do not agree whether PTPN22^620W^ is a gain-of-function or loss-of-function variant in human TCR signaling but there is compelling evidence for both views (**Figures 1B, C**) (40, 41, 47, 70, 72, 76, 79–81). PTPN22^620W^ is a loss-of-function variant in LFA-1 signaling (**Figure 2**) (32). PTPN22^620W^ has not been studied in the context of T_reg_ induction in humans, however activated T_regs_ (aT_regs_) from PTPN22^620W/W^ donors have a reduced capacity to inhibit IFNγ secretion from other T cells compared to those from PTPN22^620R/R^ donors (47).

PTPN22 is a known negative regulator of TCR signaling (**Figure 1A**) (82). To investigate the function of PTPN22 in human T cells many studies have utilized the T-cell acute lymphoblastic leukemia cell line, Jurkat (26, 28, 43). This has allowed dissection of the influence of PTPN22 on proximal TCR signaling. In Jurkat T cells, it has been shown that PTPN22 negatively regulates activation of JNK2 (26) and LCK (26), and transcriptional activity driven by NF-κB (28), CD28 response element/NF-IL2B AP-1 (43), NFAT/AP-1 (26), c-fos (26), and c-jun (26) downstream of the TCR. CRISPR/Cas9 mediated knockout of *PTPN22* in Jurkat T cells revealed that PTPN22 negatively regulates TCR-driven IL-2 and CD69 expression especially in the context of weak antigen stimulation (83). CRISPR/Cas9 mediated knockout of *PTPN22* in primary CD4^+^ T cells supports that PTPN22 is a negative regulator of TCR signaling (78). These studies also revealed how PTPN22 achieves negative regulation of TCR signaling. PTPN22 cooperates with CSK to inhibit initial TCR signaling (**Figure 1A**) (26). In resting T cells, PTPN22 is associated with CSK and upon TCR stimulation, this complex dissociates at a rate that parallels dephosphorylation of PTPN22 substrates (40).

While PTPN22^620R^ is a negative regulator of TCR signaling, the effect of the SNP on function of PTPN22^620W^ remains controversial. It is currently debated whether PTPN22^620W^ is a gain-of-function variant that reduces response to TCR stimulation (**Figure 1B**) or a loss-of-function variant that allows enhanced TCR signaling (**Figure 1C**). The most studied hypothesis is that PTPN22^620W^ is a gain-of-function variant that suppresses TCR signaling (**Figure 1B**) (40, 41, 47, 70, 72, 76, 79, 80). These studies have shown that PTPN22^620W^ reduces signaling through the TCR and is associated with significantly reduced IL-2 secretion (72, 79), calcium mobilization (72, 76, 79), and IFNγ production from CD4^+^ T cells (**Table 2**) (79). There is also evidence that the PTPN22^620W^ allotype drives enhanced skewing of CD4^+^ T cells to EOMES^+^ T_H_1 cells (47, 73). PTPN22^620W^ is also associated with reduced expression of CD25, lower proliferation, and decreased IL-10 secretion by CD4^+^ memory T cells (76, 79) (**Table 2**). In concordance with this, *in vivo* TCR stimulation in the form of a trivalent influenza vaccine resulted in reduced induction of an influenza virus-specific CD4^+^ T cell response in PTPN22^620R/W^ subjects compared to PTPN22^620R/R^ subjects (**Table 2**) (80). Another indication of reduced influenza virus-specific CD4^+^ T cell induction is the impairment of anti-flu antibody affinity maturation. Antibody affinity maturation relies on activation of CD4^+^ T follicular helper (T_FH_) cells; PTPN22^620R/W^ subjects had reduced affinity maturation compared to PTPN22^620R/R^ subjects implying they had reduced activation of T_FH_ cells or reduced activation of anti-flu B cells (80).

**Table 2:** *PTPN22* genotype influence on TCR-related phenotypes.

Phenotype	Donor genotype		
	*PTPN22^1858C/C^*	*PTPN22^1858C/T^*	*PTPN22^1858T/T^*
IFNγ production (fold change) (79)	4.34	*	1.35*
CD4^+^ memory T cells that are CD25^+^ (%) (76)	58	45	n/a
IL-10 secretion from CD4^+^ memory T cells (pg/ml) (76)	2,200	800	n/a
CD4^+^ T cell proliferation (CFSE MFI) (79)	1,834	1.8	n/a
Influenza virus-specific CD4^+^ T cells (%) (80)	0.23	n/a	0.15

*The PTPN22^1858C/T^ donors were included in the PTPN22^1858T/T^ donor group.

Studies tracing the proximal events following TCR stimulation agree that PTPN22^620W^ is a gain-of-function variant in primary T cells (**Figure 1B**). Overexpression of PTPN22^620W^ decreases NFAT/AP-1-driven luciferase transcription more than overexpression of PTPN22^620R^ (72). In primary T cells from donors with PTPN22^620W/W^ TCR stimulation resulted in lower TCRζ-chain phosphorylation and increased ERK, AKT, and PI3K p85 activation compared to PTPN22^620R/R^ donor T cells (**Table 3**) (47, 70). These studies offer a molecular mechanism for the difference in function of PTPN22^620R^ and PTPN22^620W^ centered on reduced interactions of CSK and LCK with PTPN22^620W^.

**Table 3:** *PTPN22* genotype influence on proximal TCR signaling events.

Phenotype	Donor genotype	
	*PTPN22^1858C/C^*	*PTPN22^1858T/T^*
relative TCRζ-chain phosphorylation (1 min)	100%	95%
relative ERK phosphorylation (15 min)	0%	50%
relative AKT phosphorylation (15 minutes)	40%	75%

As noted above, in resting T cells PTPN22^620R^ is associated with CSK and upon TCR stimulation this complex dissociates at a rate that parallels dephosphorylation of PTPN22 substrates (**Figure 1A**) (26, 40). Simultaneously, PTPN22 is phosphorylated at Ser^751^ by PKCα which enhances the CSK/PTPN22 interaction and restricts PTPN22 activity to allow appropriate TCR signaling (42). PTPN22^620W^ interacts with CSK to a lesser extent than PTPN22^620R^ (immunoprecipitation of PTPN22^620R^ pulls down 2.9 fold more CSK than PTPN22^620W^), and is more available to dephosphorylate PTPN22 substrates at the initiation of TCR signaling (28, 40, 41, 51). Both PTPN22^620R^ and PTPN22^620W^ are subject to phosphorylation at Ser^751^ by PKCα, however this only seems to inhibit PTPN22^620R^ activity, by enhancing its association with CSK, while it does not inhibit PTPN22^620W^ or enhance PTPN22^620W^/CSK interactions (42). Similarly, PTPN22^620R^ is associated with LCK to a greater degree than PTPN22^620W^ and this appears to be CSK-dependent (41). LCK phosphorylates PTPN22 on an inhibitory Y536 residue (41). PTPN22^620R^ has more phosphorylated Y536 residues and is less active than PTPN22^620W^ in Jurkat cells at rest and upon TCR stimulation (41). This may also explain why the *in vitro* phosphatase activity of PTPN22^620W^ is 50% higher compared to PTPN22^620R^ when the two allotypes of PTPN22 are purified from mammalian cells. When purified from insect cells, where this post-translational modification is absent, the phosphatase activity is equal among the two allotypes (41, 72). In conclusion, PTPN22^620W^ is a more potent inhibitor of TCR signaling than PTPN22^620R^ because PTPN22^620W^ is more available to interact with PTPN22 substrates due to reduced sequestration by CSK. Further, PTPN22^620W^ is more active due to reduced association with its own negative regulator, LCK, and consequent reduced phosphorylation at an inhibitory tyrosine residue (**Figure 1B**).

While evidence that PTPN22^620W^ is a gain-of-function variant remains compelling, sufficient results exists to argue that PTPN22^620W^ could be a loss-of-function variant (**Figure 1C**) (70, 81). These studies have observed that T cells from healthy PTPN22^620W/W^ donors expand more upon TCR stimulation than those from healthy PTPN22^620R/R^ donors (70). Further, when CSK is co-expressed with PTPN22^620W^ in Jurkat T Cells, higher calcium fluxes are measured than when CSK is co-expressed with PTPN22^620R^ (81). A study found that the *PTPN22^1858T^* allele enhances expression of a dominant negative isoform, PTPN22.6, that increases signaling through the TCR (**Figure 1C**) (84). The authors offered a hypothesis that reconciles human data showing that PTPN22^620W^ is a gain-of-function; PTPN22^620W^ allows chronic signaling through the TCR that drives T cell exhaustion, causing T cells from PTPN22^620R/W^ and PTPN22^620W/W^ donors to be less responsive to stimulation through the TCR—a finding reported by most studies. This is supported by evidence that expression of PD-1, a marker of T cell exhaustion, is enhanced on CD4^+^ T_eff_ and T_regs_ in healthy PTPN22^620W/W^ donors compared to healthy PTPN22^620R/R^ donors (74). Furthermore, the reduced calcium flux seen in PTPN22^620R/W^ donors was most notable in memory CD4^+^ T cells with no difference observed in naïve CD4^+^ T cells; this could indicate that the experienced population is exhausted (76). While it is not certain whether PTPN22^620W^ is a gain-of-function or loss-of-function variant in human TCR signaling, it is clear that the mouse orthologue of PTPN22^620W^, PEP^619W^, is a loss-of-function variant in mouse TCR signaling.

Data from mouse models support the role of PTPN22/PEP as a negative regulator of TCR signaling (**Figure 1A**). Overexpression of PEP in the mouse antigen specific T cell line, BI-141, reduced TCR-mediated phosphorylation of ZAP70, c-Cbl, and the CD3 ζ-chain (77). Overexpression of PEP also reduced IL-2 secretion from these cells (77). C57BL/6J mice with a genetic ablation of *Ptpn22* (B6.Cg-*Ptpn22^tm2Achn^*/J commonly referred to as C57BL/6-*Ptpn22* ^−/−^ mice) as well as NOD mice with doxycycline-induced knockdown of PEP [NOD-Tg(tetO-RNAi : *Ptpn22*,UBC-tetR,-GFP)P2Kslr commonly referred to as NOD-*Ptpn22^KD^*] starting at birth have an accumulation of effector/memory CD4^+^ and CD8^+^ T cells in secondary lymphoid organs. This phenotype is thought to be a product of increased TCR signaling in the absence of PEP (85–88). Similar to humans harboring PTPN22^620W^, PEP^619W^ knock-in C57BL/6 mice (C57BL/6-*Ptpn22* ^tm1.1Kas^ commonly referred to as C57BL/6-PEP^619W^) exhibited an expansion of CD4^+^ memory T cells compared to unaltered C57BL/6 mice that carry the PEP^619R^ allele (70, 71). In C57BL/6-PEP^619W^ mice there was also a marked expansion of the total effector/memory T cell pool and T cells from these mice exhibited increased IL-2 secretion, increased calcium mobilization, enhanced/prolonged tyrosine-phosphorylation of ZAP-70 and Lck, and increased *ex vivo* expansion of T cells compared to C57BL/6 mice (70, 71, 86). While the R619W conversion in PEP appears to be a loss-of-function variant with respect to TCR signaling (**Figure 1C**), controversy exists regarding the human autoimmunity risk allotype, PTPN22^620W^, with regard to gain-of-function or loss-of-function TCR signaling (**Figures 1B, C**). Despite this ongoing lack of clarity for PTPN22^620W^ in human TCR signaling, evidence clearly supports that PTPN22^620W^ is a loss-of-function variant in LFA-1 signaling in T cells.

### Impact of PTPN22 Allotype on LFA-1 Signaling in T Cells

LFA-1 is fundamentally important to general leukocyte trafficking. Loss of LFA-1 causes the life-threatening disease known as leukocyte adhesion deficiency (LAD) resulting in uncontrolled microbial infections (89). LFA-1 is also critical in T cell activation and migration (90). In human T cells, PTPN22 inhibits LFA-1 signaling (**Figure 2**) (32). T cells treated with PTPN22 targeting small interfering RNA (siRNA) exhibited increased ICAM-1 (LFA-1 ligand)-induced phosphorylation of LCK, ZAP70, ERK1/2, and Vav compared to cells treated with a non-targeting control (NTC) siRNA. There was also an increase in ICAM-1-induced motility in cells treated with the PTPN22 targeting siRNA (32). The autoimmune associated variant, PTPN22^620W^, is a loss-of-function variant in LFA-1 signaling (**Table 2B**). Similar to what was observed with knockdown of PTPN22, human T cells from PTPN22^620R/W^ and PTPN22^620W/W^ donors have enhanced LFA-1 induced signaling (pERK1/2 fold change over unstimulated; PTPN22^620W/W^ ~35 vs. PTPN22^620R/W^ ~25 vs. PTPN22^620R/R^ ~20) and adhesion (mean # of T cells adhered to LFA-1 coated slide at 8 min under shear flow; PTPN22^620W/W^ ~32 vs. PTPN22^620R/R^~24) compared to T cells from PTPN22^620R/R^ donors. At rest, PTPN22^620R^ and PTPN22^620W^ are aggregated near the plasma membrane of T cells. Upon engagement of ICAM-1 with LFA-1, PTPN22^620R^ leaves these aggregates, associates with CSK, and is recruited to the leading edge of migrating cells in an LCK-dependent manner where it dephosphorylates PTPN22 substrates to inhibit LFA-1 signaling (**Figure 2A**). In contrast, PTPN22^620W^ stays more clustered and is less recruited to the leading edge resulting in less PTPN22-mediated negative regulation of LFA-1 signaling (**Figure 2B**) (32).

As observed in human T cells, PEP negatively regulates mouse T cell responses to ICAM-1 stimulation (**Figure 2A**). T cells from C57BL/6-*Ptpn22* ^−/−^ mice displayed enhanced ERK1/2 phosphorylation (pERK1/2 fold change over-unstimulated; *Ptpn22* ^−/−^ ~12 vs. *Ptpn22* ^+/+^ ~8) after ICAM-1 stimulation and adhered better to ICAM-1 coated glass slides under shear flow (mean # of T cells adhered at 8 min; *Ptpn22* ^−/−^ ~55 vs. *Ptpn22* ^+/+^ ~30) compared to *Ptpn22*-intact mouse T cells (32). C57BL/6-*Ptpn22* ^−/−^ mouse T cells also had increased LFA-1 induced IFNγ secretion and were better at forming T cell-DC conjugates compared to *Ptpn22*-intact T cells (86). PEP and PTPN22 are both negative regulators of LFA-1 signaling in mice and humans (**Figure 2A**). PTPN22^620W^ is a loss-of-function variant in humans while it is not known how the PEP 619R to W conversion affects mouse LFA-1 signaling (**Figure 2B**). While the molecular mechanisms behind PTPN22’s influence on receptor-proximal signaling in T cells (i.e., activation and mobilization) are well studied, PTPN22 has also been shown to influence T_reg_ induction and function however the mechanism is less resolved.

### Treg Induction and T Cell Suppression by aTreg

PTPN22 positively regulates *in vitro* induced T_reg_ (iT_reg_) differentiation in human T cells. Primary naive T cells (CD4^+^CD127^+^CD25^−^) from PTPN22^620R/R^ healthy donors and PTPN22^620R/R^ donors with T1D were subjected to *PTPN22* knockdown with antisense oligonucleotides. Differentiation of iT_regs_ *via* treatment with IL-2/TGF-β1/αCD3/αCD28 was reduced with *PTPN22* knockdown compared to control oligonucleotide transfected cells (% of CD4 T cells that are CD25^+^FoxP3^+^; *PTPN22* knockdown resulted in ~20% iTreg vs. control ~40%) (33). No direct clinical studies have shown how PTPN22^620^ allotypes influence iT_reg_ differentiation; however, healthy PTPN22^620W/W^ donors have increased CD4^+^ T_regs_ compared to healthy PTPN22^620R/R^ donors (7.94% vs. 6.76%) implying that PTPN22^620W^ might potentiate iT_reg_ development (74, 75). Although PTPN22^620W/W^ donors have slightly more CD4^+^ T_regs_, these T_regs_ exhibit a reduced capacity to inhibit IFNγ secretion from conventional T cells compared to those from PTPN22^620R/R^ donors (47, 76).

As observed in humans, PEP also influences T_reg_ development in mice; C57BL/6-*Ptpn22* ^−/−^ mice and NOD-*Ptpn22^KD^* mice had increased numbers of T_regs_ (87, 88). Data from C57BL/6-*Ptpn22* ^−/−^ mice and NOD-*Ptpn22^KD^* mice provided evidence that deficiency of PEP reduces the TCR signal strength required for *in vitro* induction of iT_regs_ (91, 92). The iT_reg_ induction can be accomplished by stimulating naïve FoxP3-CD4^+^ T cells with a combination of agonistic anti-CD3 and anti-CD28 targeting antibodies in the presence of TGF-β (87). Lower levels of stimulation with reduced concentrations of anti-CD3 antibodies increased *in vitro* iT_reg_ induction in *Ptpn22* ^−/−^ cells compared to *Ptpn22-*intact cells. Increased concentrations of anti-CD3 resulted in elevated stimulation and decreased iT_reg_ induction in *Ptpn22* ^−/−^ cells compared to *Ptpn22-*intact cells. At levels of TCR-stimulation that drive optimal *in vitro* iT_reg_ induction in parental C57BL/6 mice, C57BL/6-*Ptpn22* ^−/−^ had reduced iT_reg_ induction (87). Much like PTPN22^620W^ humans, aged C57BL/6-PEP^619W^ mice had increased T_regs_ compared to C57BL/6 mice. However, young C57BL/6-PEP^619W^ mice exhibited no increase in T_regs_. T_regs_ from young C57BL/6-PEP^619W^ mice exhibited no differences in suppressive activity when compared to C57BL/6 mice, however T_regs_ from aged mice were not assessed. This difference may be due to the age of the mice, however, it remains to be seen if T_regs_ from older C57BL/6-PEP^619W^ mice exhibit the same defect in suppression as human T_regs_ (71). It is clear that PTPN22 plays multiple roles in human T cells and that the diabetogenic allotype of PTPN22, PTPN22^620W^, alters these roles; how might the altered function of PTPN22^620W^ in T cells impact T1D development?

### PTPN22 in T Cells and Impact on T1D

T1D is generally considered a T cell mediated disease where CD8^+^ T cells are the major islet infiltrating immune cells (93, 94). The SNP in *PTPN22*, *rs2476601*, is associated with increased risk for T1D, reduced age at onset (95), and reduced residual β cell function at diagnosis (96). This SNP affects T cell function. PTPN22 is a negative regulator of TCR (26, 28, 43, 77) and LFA-1 (32) signaling and influences aT_reg_ suppressive capacity (**Figures 1** and **2**) (47). In T cells, the effect of the T1D-risk variant PTPN22^620W^ on TCR-induced signaling is currently unresolved with data supporting both gain-of-function and loss-of-function hypotheses (40, 41, 47, 70, 72, 76, 79, 80). In contrast, PTPN22^620W^ has been characterized as a loss-of-function variant in LFA-1-induced signaling because it is not available to interact with its substrates (32). Adaptive T_regs_ (aT_regs_) from PTPN22^620W/W^ donors have reduced capacity to suppress IFNγ secretion from conventional T cells (47). The enhanced LFA-1-induced signaling and motility, and the reduced capacity of aT_regs_ to suppress IFNγ secretion from conventional T cells seen in PTPN22^620R/W^ and PTPN22^620W/W^ humans could help explain why *rs2476601* is associated with increased overall risk of T1D development. The seemingly small magnitudes of reported biochemical, phenotypic, and functional effects of PTPN22^620W^ in human T cells are surprising for a genetic variation that ranks near the top of the list for T1D genetic risk. We ask ourselves, “How could such minor fluctuations contribute to a life-threatening pathology?” The answer might lie in the thymus - the immune tissue where developing thymocytes (soon to be T cells) are exquisitely sensitive to the strength and duration of nascent TCR signaling. If PTPN22^620W^ is a gain-of-function variant in TCR signaling, the PTPN22^620W^ variant might impair the process of negative selection whereby autoreactive thymocytes are normally eliminated upon strong TCR signaling. Thus, effectively blinded to the fact that a given TCR is recognizing a self-antigen (e.g., insulin), autoreactive T cells might survive and escape into the periphery (72). More autoreactive T cells in the periphery would lead to increased autoreactive T cells surveying tissues, including the pancreas, and more opportunities for an autoimmune reaction to occur.

The alternate scenario postulates that thymic selection is more or less unaffected, and that the biologic effects of PTPN22^620W^ manifest in the periphery. If PTPN22^620W^ is a loss-of-function variant in TCR signaling, circulating T cells would be more sensitive to TCR ligation and this could explain the genesis of autoreactive T cell activation and thus autoimmunity. Both intrathymic and peripheral scenarios would be complicated by enhanced LFA-1-induced signaling (enhancing T cell migration) and reduced capacity of aT_regs_ to suppress IFNγ secretion from activated T cells that could result in enhanced T cell infiltration into tissues (i.e., islets of Langerhans) as well as secretion of more IFNγ, thus creating a more inflammatory local environment. For T cells, additional new work will be needed to understand how thymic development and intra-islet T cell function is modulated by PTPN22 variants. Is there a single dominant mechanism at fault for autoimmune risk, or is this a case of death by a thousand cuts—multiple subtle effects which alone appear innocuous but together add up to complete destruction of a vital tissue? If the story weren’t complicated enough, T cells alone might not be the culprit of T1D. Autoantibodies produced by B cells are a prevalent feature and remain the gold standard biomarker of T1D progression. While it is hypothesized that autoantibodies are not pathogenic in human T1D, B cells are thought to play an important role as antigen specific APCs. It is known that depletion of B cells with Rituximab can delay disease progression (97). Additionally, many of the other *rs2476601*-asocciated autoimmune diseases are characterized by production of autoantibodies (e.g., RA, SLE, etc.). As such, many studies have focused on the effect of the PTPN22^620R^ versus PTPN22^620W^ in human B cells.

## Regulation of B Cell Function by PTPN22 Allotypes

PTPN22 has been studied extensively in human B cells. Unlike the minor difference observed in the T cell compartment, PTPN22^620W^ has a profound impact on B cell composition (described in detail below) (76). PTPN22 also impacts signal transduction in human B cells where it functions as a negative regulator of BCR signaling and BCR-induced apoptosis (34). Because PTPN22 influences BCR signaling and BCR-induced apoptosis, it also influences the central and peripheral B cell tolerance checkpoints (27, 76, 98–100).

### Impact of PTPN22 Allotype on BCR Signaling

**Figure 3:**
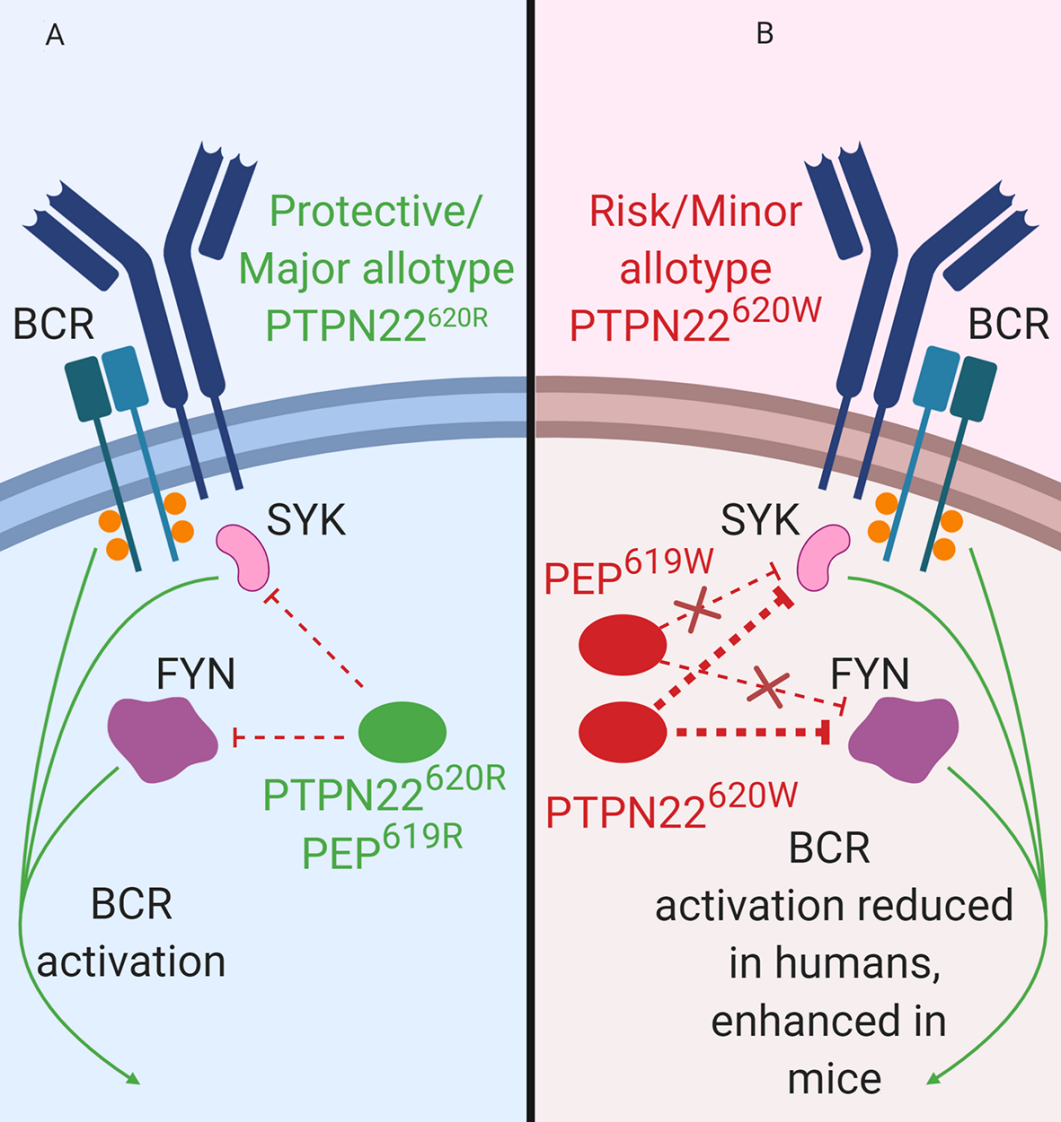
PTPN22 function in B cells. **(A)** PTPN22^620R^ and PEP^619R^ are negative regulators of BCR signaling in B cells. **(B)** PTPN22^620W^ is a gain-of-function variant with respect to BCR signaling and leads to blunted BCR signaling. PEP^619W^ is a loss-of-function variant with respect to BCR signaling and leads to enhanced BCR signaling.

PTPN22 functions to dampen BCR signaling as well as BCR-induced apoptosis (**Figure 3B**). PTPN22 is overexpressed in primary chronic B lymphocytic leukemia (CLL) cells (34). CLL cells express functional BCRs and have been characterized for ligand-dependent signaling. PTPN22 depletion in CLL cells increased soluble-αIgM (simulated strong BCR signaling) induced apoptosis (34). Knockdown of PTPN22 also resulted in increased soluble αIgM-induced phosphorylation of LYN, SYK, BLNK, PKCδ, ERK, JNK, and p38 MAPK and reduced soluble-αIgM-induced phosphorylation of AKT, GSK3, and FOXO (34). PTPN22^620W^ is a gain-of-function variant that acts to further blunt BCR signaling (**Figure 3B**). In heterozygous PTPN22^620R/W^ donors there is reduced BCR-induced calcium flux compared to PTPN22^620R/R^ donors (27, 76, 98). Heterozygous donors also had reduced phosphorylation of the BCR-proximal signaling components, SYK, PLCγ2 (MFI phospho-PLCγ2-Y759; PTPN22^620R/W^ ~700 vs. PTPN22^620R/R^ ~950), and AKT compared to PTPN22^620R/R^ donors (27, 76, 98). In PTPN22^620R/W^ donors there is also reduced total phosphorylated tyrosine in resting (% of CD27^+^ B cells that are phospho-tyrosine^+^; PTPN22^620R/W^ ~4% vs. PTPN22^620R/R^ ~8%) and BCR-activated memory B cells compared to PTPN22^620R/R^ donors (27). Inhibition of PTPN22 in B cells of PTPN22^620R/W^ donors increased SYK, PLCγ2, and AKT phosphorylation to levels equivalent to those of B cells from PTPN22^620R/R^ donors (27, 98). Signaling through the BCR can also induce B cell expansion. However, it is not clear how PTPN22^620W^ affects BCR-induced expansion of B cells; different studies have shown conflicting results (27, 70). Overall, PTPN22^620W^ is more effective at regulating BCR signaling (**Figure 3B**).

Consistent with data from human B cells, PEP is also a negative regulator of BCR signaling in mice (**Figure 3**). Silencing of PEP *via* doxycycline-induced expression of a *Ptpn22*-targeting siRNA in NOD mice (the NOD-*Ptpn22^KD^* mice) increased B cell response to anti-IgM/anti-CD40 stimulation (88). Additionally, silencing of PEP resulted in the increased proliferation, robust expression of CD25 and CD69, and elevated phosphorylation of PLCγ2 (88). These results have been replicated *via Ptpn22* knockout in other mouse strains (81, 101, 102). Unlike PTPN22^620W^ humans, C57BL/6-PEP^619W^ mice had a phenotype similar to C57BL/6-*Ptpn22* ^−/−^ mice, with increased anti-IgM induced B cell activation, increased anti-IgM induced proliferation, and increased phosphorylation of PLCγ2 compared to C57BL/6 mice (70, 71, 103). While PTPN22^620W^ is a gain-of-function variant in human BCR signaling, PEP^619W^ is a loss-of-function variant with respect to BCR signaling in mice (**Figure 3B**).

### Regulation of B Cell Gene Expression and B Cell Expression of Surface Receptors by PTPN22

PTPN22^620W^ alters gene expression and immune receptor levels in B cells. Naïve B cells from both PTPN22^620R/W^ and PTPN22^620W/W^ donors had significantly upregulated *IL4R*, *IL13R*, *IL17R*, and *IL21R* mRNA expression (genes involved in B cell proliferation/differentiation) and significantly upregulated genes in the BCR, CD40, and TLR activating pathways compared to those from PTPN22^620R/R^ donors (100). PTPN22^620W^ differentially affects expression of other genes with SNPs associated with T1D and other autoimmune diseases (*BLK, PTPN2, CD40, TRAF1, CD19, SLAM, IRF5)* (100). The surface expression of BAFFR, CD40, and SLAMF6 was enhanced in PTPN22^620R/W^ and PTPN22^620W/W^ donors compared to PTPN22^620R/R^ donors (**Table 4**) (100, 103). Naïve B cells from PTPN22^620R/W^ and PTPN22^620W/W^ donors were more responsive to CD40L stimulation with an increased percent of B cells expressing CD69 and CD25 than those from PTPN22^620R/R^ donors (100). CpG stimulation of PBMC for 4 days resulted in greater expansion of IgM+ memory B cells (CD19+CD27+IgM+) and IgM- Plasma cells (CD19+CD27^hi^IgM-) in PTPN22^620R/W^ patients with T1D compared to PTPN22^620R/R^ patients with T1D and in healthy control PTPN22^620R/W^ donors compared to healthy control PTPN22^620R/R^ donors (99). The combination of increased BAFFR, CD40, and SLAMF6 surface levels and the increased expression of *IL4R*, *IL13R*, *IL17R*, *IL21R*, as well as genes belonging to the CD40, TLR, and BCR activation pathways may explain the enhanced CpG-induced expansion of IgM+ memory B cells and IgM- Plasma cells in PBMCs seen in PTPN22^620R/W^ and PTPN22^620W/W^ donors compared to PTPN22^620R/R^ donors. Importantly, this phenomenon is present in both PTPN22^620R/W^ and PTPN22^620W/W^ donors implying that the effects of PTPN22^620W^ are either dominant or co-dominant.

**Table 4:** *PTPN22* genotype influence on B cell surface receptor expression.

Phenotype	Donor genotype					
	*PTPN22^1858C/C^*			*PTPN22^1858C/T^* and *PTPN22^1858T/T^*		
	Transitional	Naïve	IgM Memory	Transitional	Naïve	IgM Memory
BAFFR MFI	~90	~90	~90	~120	~100	~110
CD40 MFI		~190			~210
SLAMF6 MFI		~190			~200

Unlike humans, there was decreased CD40 and BAFFR surface expression on total splenocytes with decreased CD40 and BAFFR on immature B cells and increased CD40 on T2 B cells of C57BL/6-PEP^619W^ mice compared to C57BL/6 mice (103). *Tnfrsf13c* (*BAFFR*) mRNA levels were enhanced in immature B cells and *Cd40* mRNA levels were enhanced in T2 B cells of C57BL/6-PEP^619W^ mice compared to C57BL/6 mice (103). Taken together we see that PTPN22 and PEP affect expression of costimulatory molecules in B cells of both humans and mice however the effects of the R to W conversion are not consistent when comparing humans to mice.

### B Cell Tolerance Checkpoints and Composition

PTPN22^620W^ alters the central and peripheral B cell tolerance checkpoints as well as the composition of the B cell compartment in humans (76, 98–100). Central B cell tolerance is mediated *via* clonal deletion or receptor editing to remove autoreactive or polyreactive B cells from the bone marrow before they enter the periphery (e.g., spleen, blood, lymph nodes, tissues) (104). Central tolerance results in a large reduction of polyreactive and autoreactive B cells and is readily apparent when comparing the bone marrow to the spleen and blood; 40%–70% of early immature B cells are polyreactive and 50%–75% are autoreactive in the bone marrow while 5%–10% of transitional B cells are polyreactive and 30%–50% are autoreactive in the periphery (104, 105). A common method for determining if B cells are autoreactive is to assess their response to human epithelial type 2 (HEp-2) cells. HEp-2 cells express a large array of self-antigens and HEp-2 reactive B cells are considered autoreactive (106). Healthy PTPN22^620R/W^ and PTPN22^620W/W^ donors had an increased proportion of polyreactive and HEp-2-reactive new emigrant/transitional B cells (CD20^+^CD10^+^CD21^lo^IgM^hi^CD27^−^: 25%–30% of new emigrant/transitional B cells were polyreactive and ~50% were HEp-2-reactive) compared to healthy PTPN22^620R/R^ donors (8%–10% of new emigrant/transitional B cells were polyreactive and ~30% were HEp-2 reactive) (100, 107). Most studies agreed that transitional B cells (CD19^+^CD27^−^CD24^hi^CD38^hi^) were increased in healthy PTPN22^620R/W^ and PTPN22^620W/W^ donors compared to healthy PTPN22^620R/R^ donors (percentage of total B cells that are transitional; PTPN22^620W/W^ and PTPN22^620R/W^ ~5% vs. PTPN22^620R/R^ ~2.5%), although not all studies observe this effect (98, 99, 108). The increased numbers of transitional B cells and polyreactive/HEp-2-reactive new emigrant/transitional B cells in healthy PTPN22^620R/W^ and PTPN22^620W/W^ donors indicates that the central B cell tolerance checkpoint is altered by PTPN22^620W^. Ergo, the autoimmune-linked allotype allows more polyreactive and autoreactive B cells to escape central tolerance and proceed into the periphery. B cells that enter the periphery will go through another round of selection to remove or inactivate autoreactive cells.

Peripheral B cell tolerance results in anergy or clonal deletion *via* apoptosis that is dependent on caspase-3 activation and is triggered by strong signaling though the BCR (98). This results in the reduction of autoreactive peripheral B cells. There are more autoreactive transitional B cells than autoreactive naïve mature B cells due to the peripheral B cell tolerance checkpoint; 30%–50% of transitional B cells are autoreactive while 10%–30% of naïve mature B cells are autoreactive (105). To simulate strong BCR signaling in naïve B cells, anti-IgM is used to crosslink the BCRs; this is similar to encountering a multivalent self-antigen during peripheral B cell tolerance and will cause some naïve B cells to undergo apoptosis. After 12 h of anti-IgM treatment, significantly fewer naïve B cells from PTPN22^620R/W^ donors had begun the process of apoptosis by cleaving/activating caspase-3 when compared to PTPN22^620R/R^ donors (% of naïve B cells with cleaved/active caspase-3; PTPN22^620R/W^ ~10% vs. PTPN22^620R/R^ ~18%) (98). Basal levels of the anti-apoptotic protein, Bcl-2, were higher in transitional B cells from PTPN22^620R/W^ donors compared to PTPN22^620R/R^ donors (Normalized BCL-2 MFI; PTPN22^620R/W^ ~20 vs. PTPN22^620R/R^ ~12) with no alteration in the pro-apoptotic protein, Bim (98). Healthy PTPN22^620W/W^ and PTPN22^620R/W^ donors had increased frequencies of polyreactive and HEp-2-reactive mature naïve B cells (CD20^+^CD10^−^CD21^+^IgM^+^CD27^−^). In these donors ~30% of mature naïve B cells were polyreactive and ~45% were HEp-2-reactive. In contrast, healthy PTPN22^620R/R^ donors had ~10% polyreactive mature naïve B cells were and ~20% HEp-2-reactive (100). A unique subset of autoreactive anergic B cells (naïve IgD ^+^ B cells [B_ND_]: CD19^+^CD27^−^IgD^+^IgM^−^) are cells in the periphery thought to be anergic due to low chronic antigen stimulation through the BCR (109). B_ND_ cells were increased in healthy PTPN22^620R/W^ donors compared to healthy PTPN22^620R/R^ donors (% of CD19^+^ B cells that are B_ND_ cells; PTPN22^620R/W^ ~3% vs. PTPN22^620R/R^ ~2%) (98). PTPN22^620R/W^ donors had a lower percentage of memory B cells compared to PTPN22^620R/R^ donors (% of CD19^+^ B cells that are CD27^+^; PTPN22^620R/W^ ~35% vs. PTPN22^620R/R^ ~45%) (76). The reduced caspase-3 activation, increased levels of Bcl-2, increased frequencies of B_ND_ cells, HEp-2-reactive mature naïve B cells, and polyreactive mature naïve B cells, and decreased frequency of mature B cells found in PTPN22^620R/W^ and PTPN22^620W/W^ donors indicates that PTPN22^620W^ alters the peripheral B cell tolerance checkpoint (98, 100, 107). The increase in autoreactive/polyreactive new emigrant/transition B cells, all transitional B cells, B_ND_ cells, and decrease in memory B cells was also seen when comparing T1D donors regardless of genotype to healthy PTPN22^620R/R^ donors and this may represent a common B cell phenotype present in T1D patients (98, 100). Currently, it is thought that the blunting of BCR signaling by the gain-of-function PTPN22^620W^ allotype leads to reduced negative selection and is responsible for the alterations seen in central and peripheral B cell tolerance mechanisms (76, 98, 100, 107). These B cell phenotypes are observed in both patients with autoimmunity and healthy controls that encode PTPN22^620W^.

C57BL/6-*Ptpn22* ^−/−^ as well as other strains of *Ptpn22* knockout mice exhibit an altered B cell compartment. Deletion of *Ptpn22* increased age-associated B cells (ABCs), plasma cells, autoantibodies, as well as germinal center activity and size when compared to *Ptpn22*-intact mice. However, germinal center size and activity appears to be partially dependent on an alteration in T follicular helper cells (81, 101, 102). Unlike humans harboring PTPN22^620W^, alterations in the B cell compartment of the loss-of-function PEP^619W^ variant in mice is attributed to altered positive B cell selection due to enhanced BCR signaling (103). C57BL/6-PEP^619W^ mice have increased splenic transitional 1 B cells, increased age-dependent B cells (ABCs), increased class-switched B cells, increased germinal center B cells, and less mature recirculating B cells when compared to C57BL/6 mice (103). Like humans however, the enhanced positive selection leads to increased self-reactive B cells, increased autoantibody titers, and reduced apoptosis of T1 B cells in C57BL/6-PEP^619W^ when compared to C57BL/6 mice (70, 71, 103). The similarities between the B cell compartments of *Ptpn22* ^−/−^ mouse strains and C57BL/6-PEP^619W^ mice implies that PEP^619W^ is a loss-of-function variant in mice with respect to its effects on B cell positive selection while PTPN22^620W^ decreases human B cell negative selection.

While PEP^619W^ mice do not display the same central B cell tolerance phenotype as humans heterozygous or homozygous for PTPN22^620W^, immunodeficient NOD.Cg-*Prkdc^scid^.Il2rg^tm1Wjl^* (NSG) mice engrafted with human CD34^+^ hematopoietic stem cells (HSCs) from either PTPN22^620R/W^, PTPN22^620W/W^ donors, or with HSCs overexpressing PTPN22^620W^ phenocopy humans that are heterozygous or homozygous for PTPN22^620W^. These PTPN22^620W^ HSC engrafted NSG mice display an increased proportion of polyreactive and HEp-2-reactive new emigrant/transitional B cells when compared to NSG mice engrafted with HSCs from PTPN22^620R/R^ donors or HSCs overexpressing PTPN22^620R^ (100, 107). Importantly, inhibition of PTPN22 in NSG mice engrafted with PTPN22^620W^ HSCs reduced polyreactive and HEp2-reactive new emigrant B cells to the same levels as NSG mice engrafted with PTPN22^620R^ HSCs indicating that PTPN22 is the main driver of this difference (107). The increased numbers of transitional B cells and polyreactive/HEp-2-reactive new emigrant/transitional B cells in healthy PTPN22^620R/W^ and PTPN22^620W/W^ donors and in PTPN22^620W^ HSC engrafted NSG mice indicates that the central B cell tolerance checkpoint is altered by PTPN22^620W^. This alteration allows more polyreactive and autoreactive B cells to escape central tolerance and proceed into the periphery. Overall, PEP^619W^ is a loss-of-function variant in mice with respect to its effects on B cell positive selection while PTPN22^620W^ decreases human B cell negative selection; both of these alterations result in more autoreactive B cells with increased autoantibody titers.

### PTPN22 in B Cells and Impact on T1D

Autoantibodies produced by B cells are a prevalent feature of T1D and remain the gold standard biomarker of islet autoimmunity and T1D progression (110). The SNP in *PTPN22*, *rs2476601*, is associated with increased risk of persistent islet autoimmunity (i.e., autoantibodies directed against insulin, GAD65, or IA-2) (111). While the role of pathogenesis of human T1D remains controversial, the importance of B cells has been demonstrated in preclinical models and clinical trials. Depletion of B cells pauses the loss of β cell function in some patients with recent onset T1D and can prevent or reverse disease in NOD mice (97, 112). B cells are not only capable of producing antibodies, they also act as APCs to present antigen to T cells in a process called linked recognition (113). In linked recognition, B cells uptake antigen recognized by the BCR, process it, and load peptides derived from the antigen on MHC-II to present to CD4^+^ T cells (113). These responding CD4^+^ T cells must have already encountered antigen and been activated by other APCs in the periphery before they can provide T cell help to the B cells. The T cell help initiates class-switching in germinal centers, while the B cells provide co-stimulatory signals to the T cells capable of enhancing in-progress T cell responses (114). In NOD mice, it is thought that B cells primarily enhance autoreactive T cell function as APCs and through the production of pro-inflammatory cytokines (115). PTPN22^620R/W^ and PTPN22^620W/W^ donors have increased B cell surface expression of CD40, SLAMF6, and BAFFR (**Table 4**), as well as B cell mRNA expression of *IL4R*, *IL13R*, *IL17R*, *IL21R* compared to PTPN22^620R/R^ donors. PTPN22^620R^ is a negative regulator of BCR signaling and PTPN22^620W^ is a gain-of-function variant that reduces signaling through the BCR. This reduction in BCR signaling alters central and peripheral B cell tolerance allowing more autoreactive and polyreactive B cells into the periphery. The increased surface expression of CD40, SLAMF6, and BAFFR (**Table 4**), as well as B cell mRNA expression of *IL4R*, *IL13R*, *IL17R*, *IL21R* could enhance clonal expansion of B cells, differentiation into plasma cells, class switching, and cell survival in PTPN22^620R/W^ and PTPN22^620W/W^ humans (116–121). Increased SLAMF6 and CD40 expression on B cells could also enhance/prolong B cell-T cell interactions leading to more T cell and B cell activation in PTPN22^620R/W^ and PTPN22^620W/W^ humans. The combination of these phenotypes could lead to increased class switching of autoreactive B cells and increased survival of autoreactive and polyreactive B cells. These autoreactive/polyreactive B cells could go on to increase or simply sustain activation of autoreactive T cells. The increased/sustained activation of autoreactive T cells by autoreactive/polyreactive B cells could explain why *rs2476601* is associated with increased risk of persistent islet autoimmunity (111) and why treatment with a B cell depleting therapy (rituximab) can delay loss of, but not restore, the c-peptide response in patients with recent onset T1D (97).

While adaptive immune cells are integral for targeting and destroying β cells, they are not the only cells implicated in development of T1D. The innate arm of the immune system is generally required to initiate antigen-specific responses by T and B cells. Monocytes, macrophages, and dendritic cells (DCs) are all APCs capable of initiating these potent immune responses in inflammatory contexts.

## PTPN22 Allotypes in Monocytes, Macrophages, and Dendritic Cells

Monocytes, macrophages, and DCs are innate immune cells that are a part of the front-line sentinels that sense (via conserved PRRs such as TLRs and nucleic acid sensors) and eliminate invading microbes. While the function of PTPN22^620R^ and altered function of PTPN22^620W^ have been extensively examined in T and B cells, the roles of these allotypes in monocytes, macrophages, and DCs have been less studied. In human DCs and macrophages, PTPN22^620R^ is a positive regulator of TLR4-induced Type 1 interferon (T1-IFN) production while PTPN22^620W^ is less effective at driving TLR4- and TLR7/8-induced T1-IFN production (35, 122). In macrophages, PTPN22^620R^ is a positive regulator of NLRP3 inflammasome activation and PTPN22^620W^ is a gain-of-function variant leading to more NLRP3 activation and subsequent IL-1β release (36, 37). In monocytes, PTPN22^620R^ negatively regulates NOD2-induced autophagy (39) and regulates IFNγ-induced signaling (29) while PTPN22^620W^ has not been studied in the regulation of NOD2-induced autophagy or IFNγ-induced signaling. When examining the polarization of macrophages, PTPN22^620R^ is a negative regulator of IL-23/IL-12 production following M1 induction (IFNγ/LPS treatment) while PTPN22^620W^ is a gain-of-function variant that reduces IL-21/IL-12 production following M1 polarization. PTPN22^620R^ is a positive regulator of IL-10 expression following M2 induction (IL-4/IL-13 treatment) and PTPN22^620W^ does not alter this (38). As these previous studies illustrate, PTPN22 plays diverse roles in monocytes, macrophages, and DCs and the 620R to W conversion alters function in many aspects.

### TLR-Induced Type 1 Interferons

**Figure 4:**
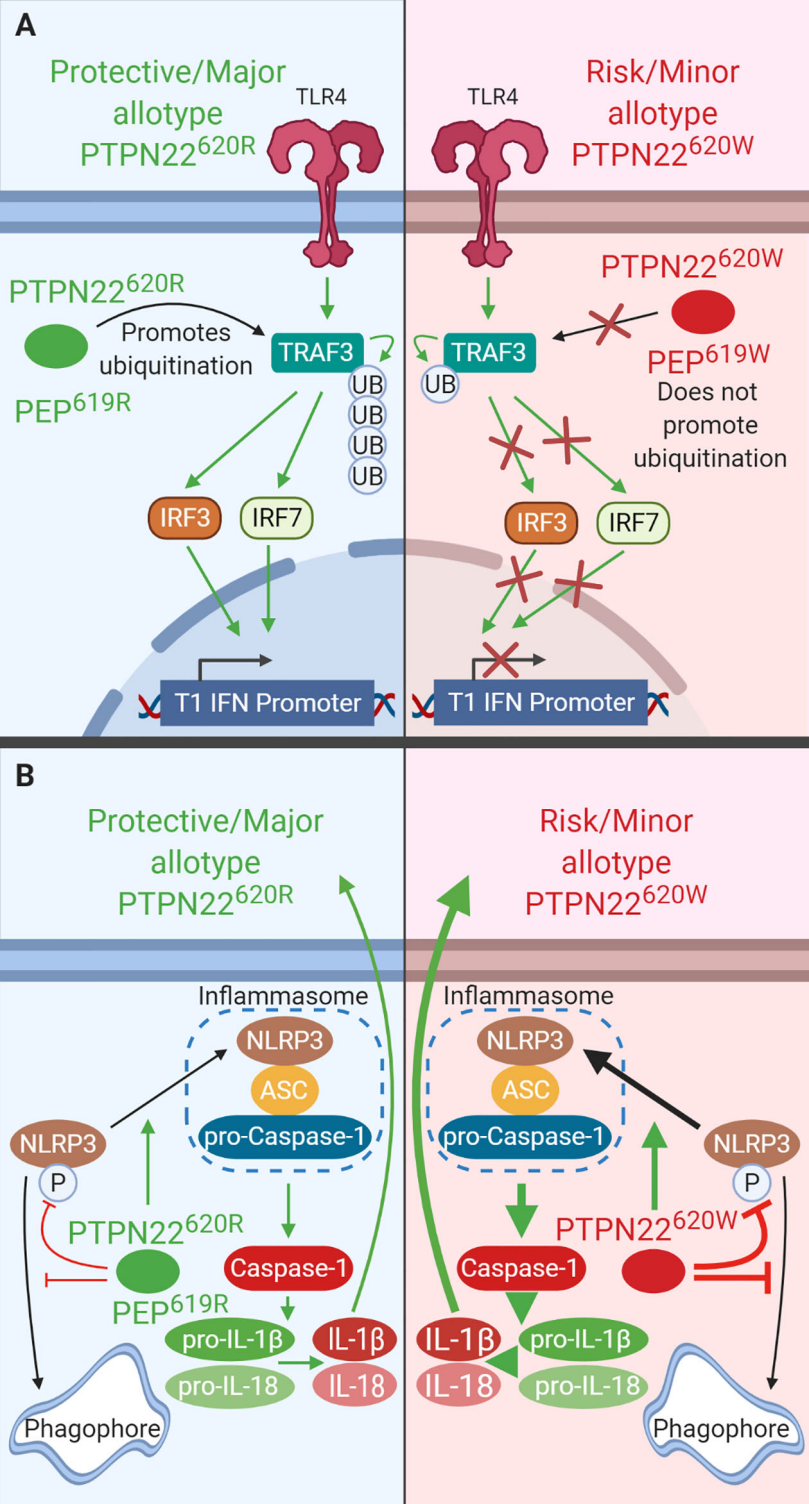
PTPN22 regulates TLR-induced T1-IFN secretion and NLRP3 inflammasome activation in macrophages and DCs. **(A)** PTPN22^620R^ and PEP^619R^ promote T1-IFN secretion in response to TLR-agonists by interacting with TRAF3 and promoting its autoubiquitination and subsequent induction of T1-IFN. PTPN22^620W^ and PEP^620W^ do not interact with TRAF3 and fail to support TLR-induced T1-IFN production. **(B)** PTPN22^620R^ and PEP^619R^ promote NLRP3 inflammasome activation by dephosphorylating NLRP3 and preventing its sequestration into the autophagosome. PTPN22^620W^ is a gain-of-function variant that has enhanced capacity to dephosphorylate NLRP3. This leads to increased NLRP3 inflammasome activation.

PTPN22^620R^ associates with TRAF3 following LPS stimulation and promotes T1-IFN production while PTPN22^620W^ does not (**Figure 4A**) (35). This effect is not limited to TLR4 stimulation, plasmacytoid dendritic cells (pDCs) from PTPN22^620W/W^ and PTPN22^620R/W^ patients with SLE have reduced IFNα production following R848 (TLR7/8 agonist) stimulation compared to PTPN22^620R/R^ patients (PTPN22^620R/W^+PTPN22^620W/W^; ~35% pDCs IFNα2+ with gMFI of ~250 vs. PTPN22^620R/R^; 45% pDCs IFNα2+ with gMFI of ~500) (122). STAT1 phosphorylation, a marker of interferon receptor signaling, is significantly reduced by about 50% in PBMCs from PTPN22^620R/W^ donors after LPS treatment when compared to PTPN22^620R/R^ donors. T1-IFN-inducible genes (*IRF7*, *MX1*, and *ISG15*) were also significantly reduced by about 50% in PBMC-derived DCs from PTPN22^620R/W^ donors compared to PTPN22^620R/R^ donors, probably due to reduced production of T1-IFNs. TRAF3 is an adaptor protein that links TLR4 and TLR7/8 signaling to induction of T1-IFNs. PTPN22 co-immunoprecipitated TRAF3 from human monocyte derived DCs (moDCs). In transgenic C57BL/6-*Ptpn22* ^−/−^ mice expressing either human PTPN22^620R^ or PTPN22^620W^, PTPN22^620R^ associated with TRAF3 and promoted its poly-ubiquitination and subsequent induction of *Ifnb1* while PTPN22^620W^ did not. C57BL/6-*Ptpn22* ^−/−^ mice expressing human PTPN22^620W^ had reduced LPS-induced T1-IFN production [~50% of *Ifnb1* from bone marrow-derived dendritic cells (BMDCs), and ~50% of *Ifnb1*/*Ifna4* from bone marrow-derived macrophages (BMMΦ)] compared to those expressing human PTPN22^620R^ (35).

Like PTPN22^620W^ in humans and transgenic mice, BMMΦ from C57BL/6-*Ptpn22* ^−/−^ mice had impaired TLR4-induced T1-IFN (*Ifnb1* and *Ifna4* mRNA production were ~50% less) and decreased TLR4- and TLR3-induced IFN-β production (~60% less) compared to WT BMMΦ (**Figure 4A**). BMMΦ from C57BL/6 mice reconstituted with PEP^227S^, a phosphatase-inactive mutant, restored TLR-induced *Ifnb1* expression indicating that the phosphatase activity of PEP is not required in this process. C57BL/6-*Ptpn22* ^−/−^ BMMΦ have reduced K63-linked polyubiquitination of TRAF3 following LPS stimulation compared to WT BMMΦ. These data are not confined to mouse BMMΦ, pDCs from C57BL/6-*Ptpn22* ^−/−^ mice and BXSB/MpJ-*Ptpn22* ^−/−^ mice had fewer pDCs making IFNα (~50% reduction) and the pDCs that were making IFNα made less than pDCs from WT mice (again ~50% reduction) (102). Also like PTPN22^620W^ humans, C57BL/6-PEP^619W^ mice had significantly reduced TLR-7-driven T1-IFN serum levels following injection of R848 compared to C57BL/6 mice (~3 ng/ml in C57BL/6-PEP^619W^ mice vs. 5 ng/ml in C57BL/6 mice) (122). The combined data from mice and humans shows that both PTPN22^620W^ and PEP^619W^ are loss-of-function variants with respect to TLR-induced T1-IFN resulting in reduced T1-IFN following TLR stimulation (**Figure 4A**) (35). TLR stimulation does not only induce T1-IFN, it is also capable of priming the NLRP3 inflammasome for subsequent activation following an inflammatory stimulus such as murmamyldipeptide (MDP), an aganoist of nucleotide-binding oligomerization domain-containing protein (NOD2) that is a component of bacterial cell walls. The role of PEP/PTPN22 allotypes in NLRP3 inflammasome may also impact autoimmunity.

### NLRP3 and IL-1β

PTPN22^620R^ positively regulates activation of NLRP3 and subsequent release of IL-1β (**Figure 4B**). PTPN22^620W^ is a gain-of-function variant that potentiates NLRP3 activity (**Figure 4B**) (36, 37). PTPN22 dephosphorylates NLPR3 at Y861 which prevents it from being sequestered into phagophores and degraded *via* autophagy (36, 37). PTPN22 knockdown in THP-1 macrophages primed with ultrapure LPS (upLPS) led to increased NLRP3 phosphorylation and increased NLRP3 sequestration in autophagosomes, with a concomitant reduction in IL-1β secretion ranging from about 50% with MDP treatment and up to 80% with monosodium urate (MSU) treatment (36, 37). In support of this, inhibiting autophagy restored IL-1β secretion from PTPN22 knockdown THP-1 cells (37). PTPN22^620W^ is a gain-of-function variant and is better able to dephosphorylate NLRP3 and prevents its sequestration into phagophores and subsequent degradation (**Figure 4B**). PTPN22^620W^ has an enhanced capacity to dephosphorylate NLRP3 in a cell free system compared to PTPN22^620R^ (36). When moDCs from PTPN22^620R/W^ donors were primed with ultrapure LPS and treated with monosodium urate (MSU) cleaved caspase-1 was increase by 500% and produced 300% more mature IL-1β compared to PTPN22^620R/R^ donors (36).

Much like THP-1 cells with PTPN22 knockdown, C57BL/6-*Ptpn22* ^−/−^ mice exhibited a 50% reduction in MDP-, MSU-, and ATP-induced IL-1β secretion from BMDCs compared to those of *Ptpn22*-competent mice and this effect was abrogated by inhibition of autophagy (**Figure 4B**) (37). This is due to the catalytic activity of PTPN22. In C57BL/6-*Ptpn22* ^−/−^ BMDCs expressing the catalytically dead human PTPN22 (PTPN22^263Q^) the same effect was observed. Similar to moDCs from PTPN22^620R/W^ donors, BMDCs from C57BL/6-PEP^619W^ mice have less NLRP3 in autophagosomes upon upLPS/MSU treatment and over 50% increased IL-1β secretion compared to C57BL/6 mice (36, 37). The same was seen when comparing BMMΦ from C57BL/6-PEP^619W^ mice with C57BL/6 mice (36). Taken together, these data demonstrate that PTPN22^620W^ and PEP^619W^ are gain-of-function variants with respect to NLRP3 dephosphorylation and enhance NLRP3-inflammasome activation and mature IL-1β release. While signaling *via* NOD2 is capable of activating the NLRP3 inflammasome following priming with LPS, it also induces autophagy and cytokine secretion.

### NOD2-Induced Autophagy and Cytokine Secretion

**Figure 5:**
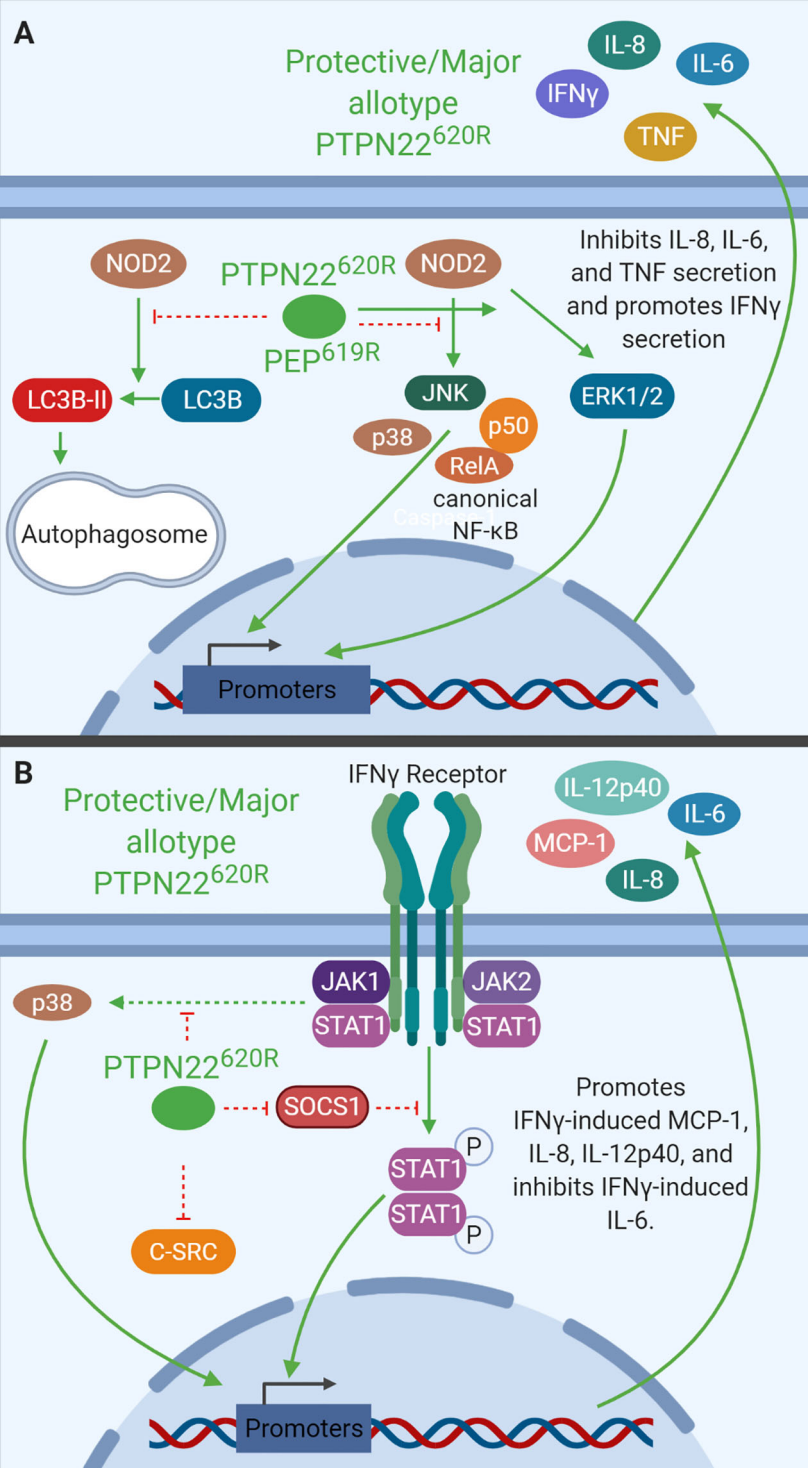
PTPN22 regulates NOD2 and IFNγ signaling in monocytes. **(A)** PTPN22^620R^ and PEP^619R^ negatively regulate NOD2-induced autophagy and NOD2-induced IL-8, IL-6, and TNF secretion while promoting NOD2-induced IFNγ secretion. **(B)** PTPN22^620R^ negatively regulates p38, SOCS1, and C-SRC activation downstream of the IFNγR. PTPN22^620R^ promotes IFNγ-induced MCP-1, IL-8, and IL-12p40, while it inhibits IFNγ-induced IL-6.

PTPN22 is a negative regulator of NOD2-induced autophagy (**Figure 5A**). Knockdown of PTPN22 *via* shRNA in THP-1 monocytes enhanced NOD2-induced LC3B-II, a cleaved and activated form of LC3B indicative of autophagosome formation. There was also a decrease in p62 protein levels consistent with enhanced autolysosome activity. Knockdown of PTPN22 *via* shRNA in THP-1 monocytes also led to enhanced JNK, p38, NF-κB-p65, and NF-κB-p50, activation downstream of NOD2 while reducing ERK activation. Enhanced NOD2-induced *IL-6* and *TNF* mRNA expression and IL-6, IL-8, and TNF secretion were also seen with PTPN22 knockdown (36, 39). In addition, the reduction in PTPN22 resulted in decreased NOD2-induced ICAM1, NOD2, T-bet, and IFN-γ mRNA expression as well as reduced IFN-γ secretion (39). Interestingly, the variant in PTPN22 is associated with reduced risk of Crohn’s disease while loss-of-function mutations in NOD2 are associated with increased risk of Crohn’s disease (123, 124). This could indicate that the T1D-risk allotype (PTPN22^620W^) enhances NOD2 activity to suppress gastrointestinal pathology, however, more studies are necessary to clarify how PTPN22^620W^ alters NOD2 response compared to PTPN22^620R^. These PTPN22 knockdown studies indicate that PTPN22 negatively regulates NOD2-induced autophagy, IL-6, IL-8, and TNF production while positively regulating NOD2-induced ICAM1, NOD2, and IFN-γ production.

Like PTPN22 knockdown in THP-1 cells, C57BL/6-*Ptpn22* ^−/−^ mice demonstrate that PEP is a negative regulator of NOD2-induced cytokine secretion in BMDCs of mice (**Figure 5A**). BMDCs from *Ptpn22* ^−/−^ mice treated with MDP had increased p38, NF-κB p65, and NF-κB p50 phosphorylation, and decreased ERK phosphorylation compared to *Ptpn22*-competent BMDCs. MDP-treated BMDCs from *Ptpn22* ^−/−^ mice had increased levels of *IL6* and *TNF* but decreased levels of *NOD2*, *ICAM-1*, and *IFNγ* mRNA compared to *Ptpn22* competent BMDCs (39). MDP-treated *Ptpn22* ^−/−^ BMDCs had enhanced IL-6, IL-8, and TNF secretion compared to *Ptpn22*-intact BMDCs (39). These data closely mirror data from PTPN22 knockdown THP-1 cells and demonstrate that PTPN22^620R^ and PEP^619R^ are negative regulators of NOD2-induced autophagy and cytokine secretion (**Figure 5A**). PTPN22 does not only influence signaling downstream of TLRs and other pattern recognition receptors in monocytes, macrophages, and DCs, it also influences cytokine secretion and signaling in response to IFNγ.

### IFNγ Receptor Signaling

PTPN22 regulates IFN-γ receptor (IFNγR) signaling in human monocytes (**Figure 5B**). PTPN22 knockdown in THP-1 monocytes followed by treatment with IFNγ induced increased SOCS1 phosphorylation and activity and reduced protein levels of SOCS3 compared to control siRNA transfected cells. PTPN22 pulls down with SOCS1, suggesting that PTPN22 may be responsible for dephosphorylating and inactivating SOCS1 when it is present. In agreement with this, PTPN22 knockdown reduced activation (phosphorylation) of known SOCS1 targets, Jak1, STAT1, and STAT3 in response to IFNγ. It also reduced subsequent production of ICAM1 (~70% reduced), NOD2 (~15% reduced), and T-bet mRNA (~40% reduced) when compared to control siRNA transfected cells. Knockdown of PTPN22 also decreased IFNγ-induced MCP-1 (~70% reduced), IL-8 (~50% less), and IL12p40 (~75% reduced) secretion (29). These data indicate that PTPN22 is a positive regulator of STAT1 and STAT3 activation following IFNγ treatment. Activation of STAT1 and subsequent gene induction is the most well characterized portion of IFNγR signaling, however, the signaling cascade activated by the IFNγR includes many other signaling molecules. Treatment with IFNγ also induces signaling *via* p38 MAPK and Src. Upon knockdown of PTPN22 in THP-1 monocytes, IFNγ-induced p38 MAPK activation and subsequent IL-6 mRNA expression and protein production were enhanced compared to control siRNA transfected cells. This suggests that PTPN22 is negatively regulating p38 MAPK activation downstream of the IFNγR. It is unknown how PTPN22 regulates p38 MAPK activation downstream of the IFNγR, however, there are several plausible targets. Current literature indicates that p38 MAPK is activated by the IFNγR *via* a signaling cascade involving JAK2, Pyk2, MEKK4, MEK6, and finally p38 MAPK (125, 126). Pyk2, MEKK4, and p38 MAPK are attractive potential targets of PTPN22 because they are all activated by phosphorylation on a tyrosine residue. At this time, more targeted research is necessary to define the PTPN22 target(s) in this pathway. Similarly, PTPN22 knockdown induced basal Src phosphorylation that increased after IFNγ treatment; however, in control siRNA transfected cells there was no basal Src phosphorylation nor was there IFNγ-induced Src phosphorylation. This indicates that PTPN22 negatively regulates basal Src activation and IFNγR-induced Src activation (29). While PTPN22 influences response to IFNγ treatment alone it also influences macrophage cytokine secretion following polarization in response to IFNγ/LPS or IL-4/IL-13 treatment.

### Macrophage Polarization

**Figure 6:**
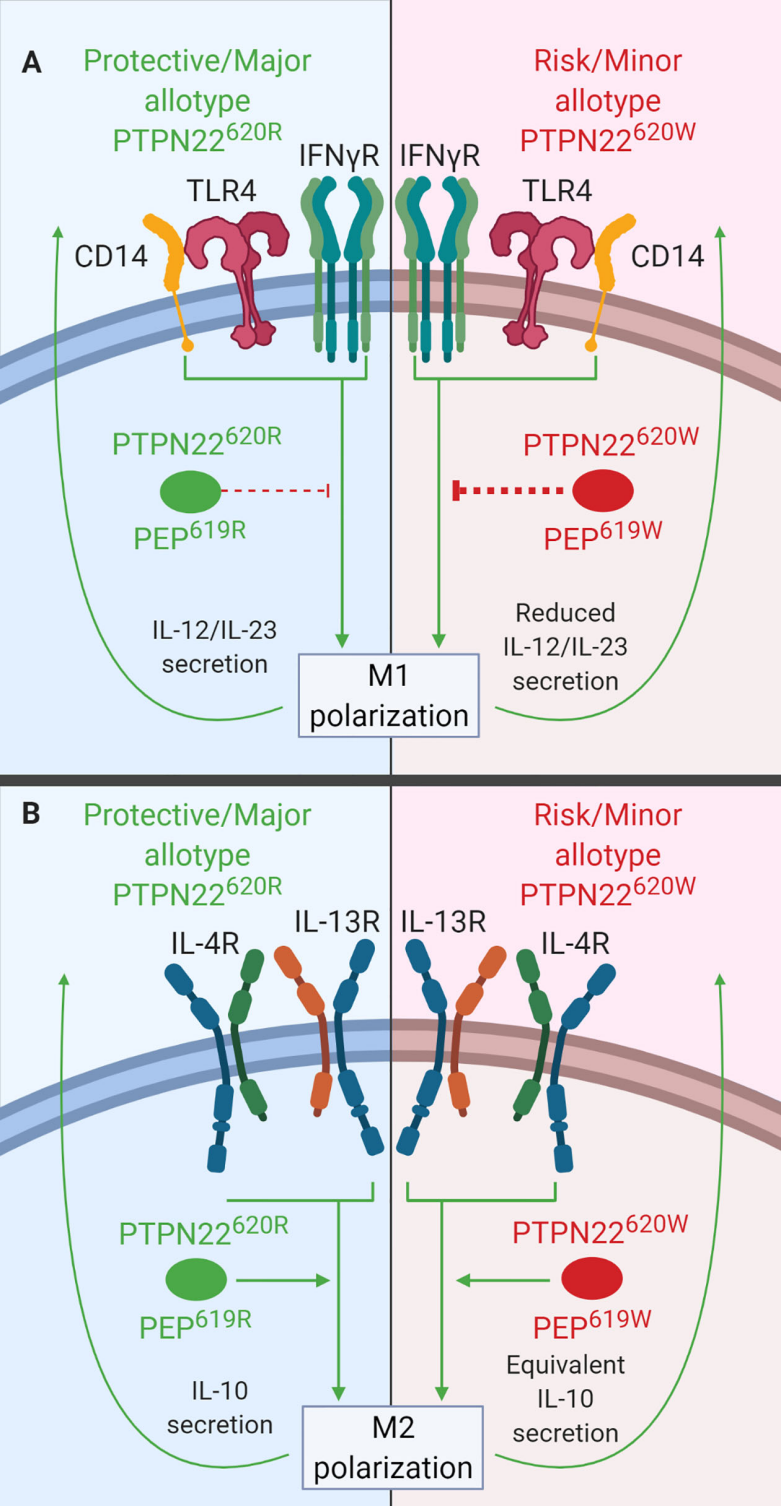
PTPN22 regulates macrophage polarization. **(A)** PTPN22^620R^ and PEP^619R^ inhibit cytokine secretion from M1 macrophages. Upon M1 polarization of PTPN22^620W^ or PEP^619W^ macrophages, there is more PTPN22^620W^ and PEP^619W^ present and the enhanced expression leads to an increased capacity to inhibit cytokine secretion from M1 macrophages. **(B)** PTPN22^620R^ and PTPN22^620W^ promote cytokine secretion from M2 macrophages equivalently.

In primary MDMs, PTPN22 is a negative regulator of IL-12 and IL-23 production following M1 polarization (**Figure 6A**) and a positive regulator of IL-10 production following M2 polarization (**Figure 6B**). PTPN22 knockdown in MDMs led to increased IL-23 (~60% more) and IL-12 (~30% more) secretion upon IFNγ/LPS treatment (M1 polarization) and decreased IL-10 expression (~50% less) following IL-4/IL-13 treatment (M2 polarization). PTPN22^620W^ appears to be a gain-of-function negative regulator of IL-12 and IL-23 production following M1 polarization (**Figure 6A**). M1 polarized macrophages from PTPN22^620W/W^ donors expressed significantly less IL-12, IL-1β, and IL-6 than those from PTPN22^620R/R^ donors. It is thought that this gain-of-function phenotype is due to enhanced expression of PTPN22^620W^ upon M1 polarization. M1 polarized macrophages from PTPN22^620W/W^ donors expressed significantly more PTPN22 than those from PTPN22^620R/R^ donors. PTPN22^620W^ and PTPN22^620R^ are comparable positive regulators of M2 polarization with no differences in IL-10 expression following IL-4/IL-13 treatment (**Figure 6B**) (38).

Like PTPN22 knockdown in human MDMs, splenic macrophages from C57BL/6-*Ptpn22* ^−/−^ mice had increased expression of IL-23 (~200%) and IL-12 (~250%) following M1 polarization (**Figure 6A**) and decreased expression of IL-10 (~50%) following M2 polarization (**Figure 6B**) compared to those from *Ptpn22*-intact mice (38). These *Ptpn22* ^−/−^ splenic macrophages had increased NF-κB activity (~200%) compared to *Ptpn22*-intact macrophages and this could explain the increase in LPS/IFNγ-induced IL-12 and IL-23. Splenic macrophages from C57BL/6-*Ptpn22* ^−/−^ mice reconstituted *in vitro* with PEP^619R^ or PEP^619W^ and then polarized to M1 or M2 macrophages had no difference in gene expression. If the level of PEP expression is important in mouse macrophages like the level of PTPN22 expression is in human MDMs, then reconstituting macrophages with the same amount or PEP^619R^ and PEP^619W^ would not capture the effects seen in human MDMs where PTPN22^620W^ and PTPN22^620R^ expression levels are different (38). Like human PTPN22^620W^ M1 macrophages, M1 peritoneal macrophages from C57BL/6-PEP^619W^ mice had lower mRNA levels for the M1 genes, iNOS (~50 fold less) and TNF (~2 fold less), than those from WT mice (127). Overall, these data indicate that PTPN22^620W^ and PEP^619W^ are gain-of-function negative regulators of macrophage cytokine secretion following M1 polarization due to increased PTPN22 expression (**Figure 6A**). PTPN22 has multiple roles in macrophage polarization and in fact influences diverse functions in monocytes, macrophages, and DCs. The T1D-associated variant of PTPN22, PTPN22^620W^, influences a large number of these functions and these cellular phenotypes could contribute to the pathogenesis of T1D.

### PTPN22 in Monocytes, Macrophages, and DCs and Impact on T1D

Monocytes, macrophages, and DCs are APCs that are all capable of initiating and enhancing adaptive immune responses. The precipitating events that lead to loss of tolerance and the development of T1D are unknown; be it physiological β cell death, viral infection, bacterial infection, or some other initiating event, monocytes, macrophages, and DCs are the cells most likely to sense β cell death/inflammation and initiate the adaptive immune response. After APCs trigger the adaptive immune response, these cells enhance and support the ongoing immune response against β cells. In APCs, PTPN22^620R^ plays a role in signaling downstream of many PRRs [i.e., TLR4 (35), TLR7/8 (122), NOD2 (36, 37, 39)], cytokine receptors [i.e., IL-4R/IL-13R (38), and IFNγR (29, 128)]. PTPN22^620W^ enhances NLRP3 activation and subsequent IL-1β release following priming *via* TLR4 (LPS) and treatment with a NOD2 agonists (MDP) while dampening the T1-IFN response following TLR4/7/8 stimulation. The combination of these phenotypes renders APCs from PTPN22^620R/W^ and PTPN22^620W/W^ humans more sensitive to NLRP3 activation while dampening their ability to produce T1-IFNs in response to PRR signaling. IL-1β enhances naïve and memory CD4 T cell expansion and this could in turn exacerbate activation of autoreactive CD4 T cells during the initiation of T1D (129). T1-IFNs enhance CD8 T cell activation and support activated T cell survival and are considered a major feature of the diabetic islet microenvironment where they enhance expression of MHC-I on β cells and expression of T cell chemoattractants (e.g., CXCL10) (130–132). Importantly, the T1-IFN phenotype results in a reduction of T1-IFN and not a complete loss. This might reduce the induction of MHC-I and T cell chemoattractants, however, it would not ablate them and in a genetically predisposed individual this may still be more than sufficient to help initiate and sustain T1D especially in combination with enhanced IL-1β production.

## Neutrophils

**Figure 7:**
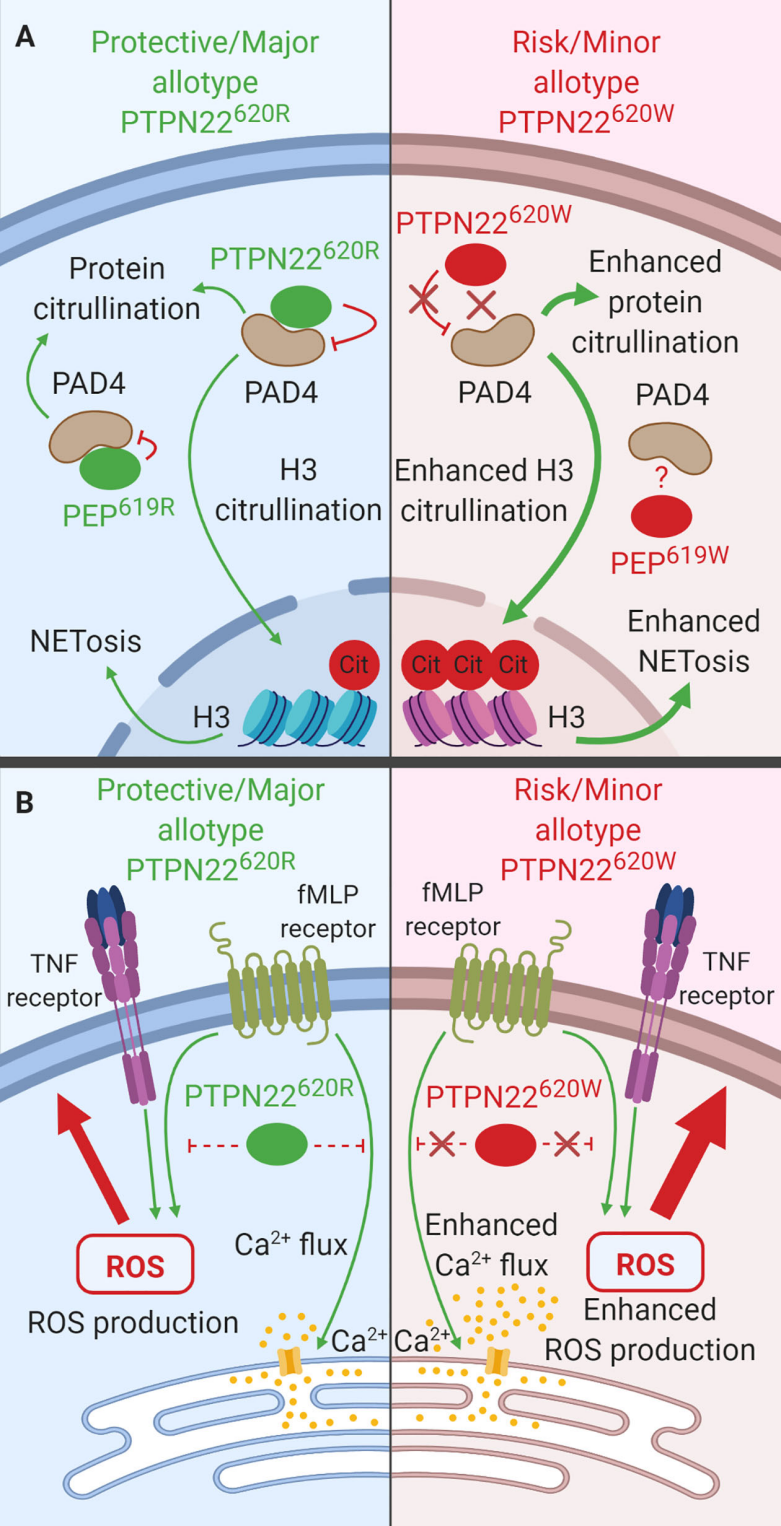
The function of PTPN22 in Neutrophils. **(A)** PTPN22^620R^ and PEP^619R^ are negative regulators of PAD4 activation and subsequent citrullination of target proteins in neutrophils. PTPN22^620W^ is a loss-of-function variant that potentiates PAD4 activation and citrullination of PAD4 targets. **(B)** PTPN22^620R^ is a negative regulator of fMLP induced calcium flux and ROS production while PTPN22^620W^ a loss-of-function variant that results in enhanced fMLP-induced calcium flux and ROS.

While neutrophils are not essential for T1D pathology (133, 134), they do play a role in other *rs2476601*-associated autoimmune diseases (e.g., RA, SLE). Thus, it is paramount to consider how PTPN22 influences neutrophil function (135). Importantly, PTPN22 is expressed in neutrophils and PTPN22^620^ allotype influences neutrophil function. This section will review what is known about the function of PTPN22^620R^ and PTPN22^620W^ in human neutrophils. PTPN22 protein level does not vary when comparing neutrophils from PTPN22^620R/R^ and PTPN22^620R/W^ donors; however, at time of writing, PTPN22^620W/W^ donors have not been assessed for neutrophil PTPN22 content (30). In human neutrophils, PTPN22 plays a role in protein citrullination (30), neutrophil extracellular trap formation (NETosis) (**Figure 7A**) (30), transmigration across inflamed endothelium (31), and response to N-formyl- Methionine-Leucine-Phenylalanine (fMLP) (**Figure 7B**) (31). PTPN22^620R^ has been shown to interact with PAD4 in human neutrophils and is a negative regulator of PAD4 activity and NETosis while PTPN22^620W^ is a loss-of-function variant in this process (**Figure 7A**) (30). PTPN22^620W^ potentiates neutrophil calcium flux and ROS production in response to fMLP stimulation (**Figure 7B**) as well as transmigration across inflamed epithelium when compared to PTPN22^620R^ (31).

### Protein Citrullination and NETosis

PTPN22 is a negative regulator of protein citrullination and NETosis and PTPN22^620W^ is a loss-of-function variant (30) (**Figure 7A**). Neutrophils from heterozygous PTPN22^620R/W^ donors displayed a hypercitrullinated protein profile (~4 fold more in PTPN22^620R/W^ neutrophils), they had enhanced citrullination of histone H3, a marker of NETosis (~5 fold more in PTPN22^620R/W^ neutrophils), and they were more prone to NETosis (3%–15% of PTPN22^620R/W^ neutrophils vs. ~2% of PTPN22^620R/R^ neutrophils) compared to those from PTPN22^620R/R^ donors (30, 136). PAD4 co-immunoprecipitated PTPN22 in human neutrophils and PTPN22 allotype influences this interaction; there is a significantly decreased amount of PTPN22 co-immunoprecipitated with PAD4 in heterozygous PTPN22^620R/W^ donors when compared to PTPN22^620R/R^ donors (~66% decreased). The total PTPN22 protein level was the same between donors implying that PTPN22^620R^ interacts with PAD4 more than PTPN22^620W^. In C57BL/6-*Ptpn22* ^−/−^ mouse macrophages transfected with human PTPN22^620R^ or PTPN22^620W^ expressing constructs, PTPN22^620R^ but not PTPN22^620W^ reduced protein citrullination and co-immunoprecipitated with PAD4 further supporting the lack of association of PTPN22^620W^ with PAD4 (30).

Much like in human neutrophils, PEP in C57BL/6 mouse neutrophils interacts with PAD-4. PEP co-immunoprecipitated with PAD-4. The absence of PEP in C57BL/6 mice enhanced protein citrullination by approximately 100%; however, the enhanced protein citrullination was abrogated in the presence of a catalytically dead PEP indicating that the catalytic activity of PEP is not involved in this process. Unlike in humans, PEP does not specifically impact histone H3 citrullination or NETosis in mouse neutrophils (30). Taken together, these data indicate that PTPN22^620R^ is a negative regulator of protein citrullination and NETosis in human neutrophils and PTPN22^620W^ is a loss-of-function variant (**Figure 7A**).

### Transmigration, ROS Production, and Calcium Flux

PTPN22 plays a role in transmigration across inflamed endothelium, as well as the response to fMLP, a highly chemotactic n-formylated oligopeptide actively released by invading bacteria or passively released by mitochondria of dying host cells (31, 137, 138). Significantly more neutrophils from PTPN22^620R/W^ donors transmigrate across inflamed (TNF treated) endothelium over 2 min than those from PTPN22^620R/R^ donors (PTPN22^620R/W^ = 43 ± 9% vs. PTPN22^620R/R^ = 24 ± 4%). Stimulation of neutrophils from healthy PTPN22^620R/W^ donors with fMLP resulted in increased calcium flux compared to neutrophils from healthy PTPN22^620R/R^ donors (PTPN22^620R/W^ = 0.28 ± 0.02 vs. PTPN22^620R/R^ = 0.24 ± 0.02 Indo-1 ratio). Priming of neutrophils from healthy PTPN22^620R/W^ donors with TNF followed by stimulation with fMLP resulted in significantly increased ROS production (4-fold increase) compared to PTPN22^620R/R^ donors (**Figure 7B**) (31).

Unlike in humans, PEP does not appear to play a role in transmigration across inflamed endothelium or the response to fMLP in C57BL/6 mice. *Ptpn22* ^−/−^ and *Ptpn22*-intact mouse neutrophils migrated across TNF-treated endothelium at the same rate (139). *Ptpn22* ^−/−^ and *PTPN22*-intact neutrophils produce similar amounts of ROS in response to fMLF (also called fMLP) and PMA stimulation, however, they were not primed with TNF like the human neutrophils which may explain why there was no difference in ROS production. Neutrophils from C57BL/6-*Ptpn22* ^−/−^ mice did however exhibit decreased ROS production (~50% reduced) and degranulation (~25% reduced) in response to FcγR and integrin stimulation compared to neutrophils from C57BL/6 mice. These pathways have not been investigated in the context of PTPN22 in humans (139). In human neutrophils, PTPN22^620W^ enhances transmigration across inflamed endothelium, calcium flux in response to fMLP stimulation, and ROS production in response to TNF priming followed by fMLP stimulation.

### PTPN22 in Neutrophils and Impact on T1D

Current data indicates that neutrophils most likely do not play a direct role in the pathogenesis of T1D in humans (133, 134) and it is apparent that they do not influence pathogenesis in NOD mice; depletion of neutrophils starting at 4 weeks of age does not impact development of T1D in NOD mice (134). While data indicate that neutrophils do not play a role in human T1D pathogenesis, many neutrophil products (e.g., ROS, NETs, cytokines) are capable of damaging tissues, including pancreatic β cells (140). Neutrophils from PTPN22^620R/W^ donors had enhanced calcium flux and ROS production in response TNF priming followed by treatment with fMLP compared to those from PTPN22^620R/R^ donors. These PTPN22^620R/W^ neutrophils also transmigrated across TNF-inflamed epithelium faster than their PTPN22^620R/R^ counterparts, displayed enhanced protein citrullination, and were more prone to NETosis (30, 31, 136). The combined effects of these phenotypes mean that PTPN22^620R/W^ and PTPN22^620W/W^ patients with T1D could display enhanced neutrophil accumulation in the exocrine pancreas due to enhanced transmigration across inflamed epithelium and increased frequency of these infiltrating neutrophils releasing NETs and producing high amount of ROS. More studies need to be undertaken to understand if neutrophils participate in the pathogenesis of human T1D and if the influence of PTPN22^620^ allotype effects their participation. Overall, it is clear that PTPN22 plays diverse roles in many cell types that have the potential to influence the pathogenesis of T1D.

## Conclusions

PTPN22 acts as a negative regulator of TCR and BCR signaling by preventing weak TCR/BCR ligation from activating T cells or B cells. In addition, PTPN22 functions in diverse signaling pathways in leukocytes. This phosphatase downregulates signaling in the NOD2, IFNγ/LPS, IFNγR, and fMLP receptor signaling pathways. Conversely, PTPN22 positively regulates NLRP3 inflammasome activation, TLR4/7/8 induction of T1-IFN secretion, PAD4 activation, and IL-4/IL-13 signaling. There are several rare genetic variants of *PTPN22* in humans that are associated with increased or decreased risk of autoimmune diseases. Also, *rs2476601* marks the PTPN22^R620W^ variant that is associated with increased risk for T1D and many other autoimmune diseases (28, 51–59). The 620R->W conversion creates a gain-of-function variant that suppresses TCR/BCR signaling and impacts autoimmunity by increasing the number of autoreactive T cells and B cells that escape central tolerance. Similarly, *rs56048322*, marks the variant, PTPN22^K750N^, and is associated with increased risk of T1D. The 750K->N conversion induces alternative splicing of PTPN22 that results in a novel isoform that competes with other PTPN22 isoforms for CSK binding causing T cell hyporesponsiveness and, like *rs2476601*, could allow more autoreactive T cells to escape central tolerance (48). In contrast, *rs33996649*, encodes the variant, PTPN22^R263Q^, which has diminished phosphatase activity and reduces risk for SLE and RA possibly by enhancing T cell central tolerance (49, 50).

The mouse orthologue of *PTPN22*, *Ptpn22* encoding PEP, plays similar roles to human PTPN22 and is even included in one of the *insulin-dependent diabetes* (*Idd*) intervals, *Idd18.2* (141). While rodent models, especially the NOD mouse, have been integral to furthering our understanding of T1D, the analogous mutation to PTPN22^R620W^, PEP^R619W^, is not naturally present in NOD and does not induce the same phenotype as observed in humans. This is not entirely surprising, PTPN22 and PEP are two of the most divergent phosphatase orthologues between humans and mice (38, 142). PTPN22 and PEP share 70% amino acid identity overall and only 61% amino acid identity in the c-terminal domain, where *rs2476601* lies (38, 45, 51, 142).

PTPN22 is also expressed in NK cells, monocytes, macrophages, DCs, and neutrophils where it influences diverse signaling pathways (28, 68). The expression of PTPN22 in APCs adds another layer of possible confounding factors when interpretting data in TCR and BCR signaling due to the fact that APCs directly influence T cell and B cell activation. Data describing the influence of PTPN22 on interactions of APCs with T cells in humans is lacking, but there are hints in both human and mouse data that can inform future studies. T cell/macrophage interactions are largely mediated by IFNγ/IFNγR and CD40L/CD40 in an antigen-dependent context (143, 144). PTPN22 knockdown in THP-1 monocytes had diverse effects on the IFNγR signaling pathways. PTPN22 knockdown increased activation of SOCS1, and predictably led to lower activation of JAK1, STAT1, and STAT3, known SOCS1 targets, as well as lower mRNA expression of ICAM-1, NOD2, and T-bet. PTPN22 knockdown also enhanced IFNγR-induced p38 activation and subsequent IL-6 mRNA and protein expression (29). There have not been any studies of the impact of PTPN22 on human CD40 signaling however heterozygous and homozygous PTPN22^620W^ donors have increased CD40 expression on their immature B cells compared to PTPN22^620R^ donors promoting speculation that CD40 signaling would be enhanced in these cells (100, 103). All of these data emphasize the need to elucidate how PTPN22^620W^ influences human macrophage and DC expression of CD40, MHC-II, CD80 and CD86.

At this time, there are more questions than answers pertaining to the influence of *rs2476601* on TCR signaling and the interface of the innate and adaptive arms of the immune system. More studies aimed at illucidating the impact PTPN22^620W^ has on TCR signaling and innate immune cell/adaptive immune cell interactions and crosstalk in humans need to be undertaken, especially in light of the conflicting data between mouse PEP^619W^ and human PTPN22^620W^ studies, to answer these questions.

## Author Contributions

LA, MW, and CM wrote and edited the manuscript. All authors contributed to the article and approved the submitted version.

## Funding 

This work was supported by research grants from the National Institutes of Health UC4 DK104194 (CM), UG3 DK122638 (CM), P01 AI042288 (CM, MW), T32 DK108736 (LA), and the Sebastian Family Endowment for Diabetes Research.

## Conflict of Interest

MW is currently employed by Century Therapeutics.

The remaining authors declare that this work was conducted in the absence of any commercial or financial relationships that could be construed as a potential conflict of interest.

## Abbreviations

ABCs, Age-associated B cells; APCs, Antigen presenting cells; aT_reg_, Activated T_reg_; BCR, B cell Receptor; BMDC, Bone marrow-derived dendritic cell; BMMΦ, Bone marrow-derived macrophage; BND cells, Naïve IgD+ B cells; CLL, Chronic B lymphocytic leukemia; CSK, C-src tyrosine kinase; DCs, Dendritic cells; fMLF, N-formyl-Methionine-Leucine-Phenylalanine; fMLP, N-formyl-Methionine-Leucine-Phenylalanine; Hep-2 cells, Human epithelial type 2 cells; HLA, Human Leukocyte Antigen; HSCs, CD34+ hematopoietic stem cells; Idd intervals, Insulin-depending diabetes intervals; IFNγR, Interferon gamma receptor; iT_reg_, Induced T_reg_; LAD, Leukocyte adhesion deficiency; MDM, Monocyte-derived macrophage; MDP, Muramyldipeptide; MHC, Major Histocompatibility Complex; moDC, Monocyte-derived dendritic cell; MSU, Monosodium urate; NETosis, Neutrophil extracellular trap formation; NOD2, Nucleotide-binding oligomerization domain-containing protein; NTC siRNA, Non-targeting control siRNA; PBMC, Peripheral blood mononuclear cells; pDC, Plasmacytoid dendritic cell; PRR, Pattern recognition receptor; PTPN22, Protein tyrosine phosphatase non-receptor type 22; RA, Rheumatoid arthritis; ROS, Reactive oxygen species; siRNA, Small interfering RNA; SLE, Systemic lupus erythematosus; SNP, Single nucleotide polymorphism; T1D, Type 1 Diabetes; T1-IFN, Type 1 interferon; TCR, T cell receptor; TLR, Toll-like receptor; T_reg_, T regulatory cell; upLPS, Ultrapure LPS.
